# Iodine/Chlorine Multi‐Electron Conversion Realizes High Energy Density Zinc‐Iodine Batteries

**DOI:** 10.1002/advs.202410988

**Published:** 2024-11-05

**Authors:** Jiajin Zhao, Yan Chen, Mengyan Zhang, Ziqi An, Binbin Nian, Wenfeng Wang, Hao Wu, Shumin Han, Yuan Li, Lu Zhang

**Affiliations:** ^1^ College of Environment and Chemical Engineering Hebei Key Laboratory of Applied Chemistry State Key Laboratory of Metastable Materials Science and Technology Yanshan University Qinhuangdao 066004 P. R. China; ^2^ State Key Laboratory of Materials‐Oriented Chemical Engineering School of Pharmaceutical Sciences Nanjing Tech University Nanjing Jiangsu 210009 P. R. China

**Keywords:** deep eutectic solvent electrolyte, ethylene glycol, high energy density, iodine, zinc battery

## Abstract

Aqueous zinc‐iodine (Zn‐I_2_) batteries are promising energy storage devices; however, the conventional single‐electron reaction potential and energy density of iodine cathode are inadequate for practical applications. Activation of high‐valence iodine cathode reactions has evoked a compelling direction to developing high‐voltage zinc‐iodine batteries. Herein, ethylene glycol (EG) is proposed as a co‐solvent in a water‐in‐deep eutectic solvent (WiDES) electrolyte, enabling significant utilization of two‐electron‐transfer I^+^/I^0^/I^−^ reactions and facilitating an additional reversibility of Cl^0^/Cl^−^ redox reaction. Spectroscopic characterizations and calculations analyses reveal that EG integrates into the Zn^2+^ solvation structure as a hydrogen‐bond donor, competitively binding O atoms in H_2_O, which triggers a transition from water‐rich to water‐poor clusters of Zn^2+^, effectively disrupting the H_2_O hydrogen‐bond network. Consequently, the aqueous Zn‐I_2_ cell achieves an exceptional capacity of 987 mAh g_I2_^−1^ with an energy density of 1278 Wh kg_I2_^−1^, marking an enhancement of ≈300 mAh g^−1^ compared to electrolyte devoid of EG, and enhancing the Coulombic efficiency (CE) from 68.2% to 98.7%. Moreover, the pouch cell exhibits 3.72 mAh cm^−2^ capacity with an energy density of 4.52 mWh cm^−2^, exhibiting robust cycling stability. Overall, this work contributes to the further development of high‐valence and high‐capacity aqueous Zn‐I_2_ batteries.

## Graphical abstract

A novel water‐in‐deep eutectic solvent electrolyte uses ethylene glycol as a co‐solvent to transform water‐rich clusters into water‐poor clusters, enabling the cathode to achieve the two‐electron conversion of I^+^/I^0^/I^–^ and the conversion of Cl^0^/Cl^–^. The aqueous Zn‐I_2_ cell achieves an exceptional capacity and energy density with high Coulombic efficiency.

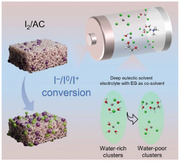

## 1 Introduction

Aqueous zinc‐based batteries possess the potential to revolutionize the future of storage batteries owing to their exceptional safety, low cost, sustainability, and high specific capacity (820 mAh g^−1^) afforded by the zinc anode, which operates at an optimal potential (0.76 V versus Standard Hydrogen Electrode, SHE).^[^ ^1^, ^2^, ^3^, ^4^, ^5^ ^]^ However, traditional ion storage methods that rely on intercalation into crystalline cathode materials, such as MnO_2_ and V_2_O_5_ encounter challenges, including approaching their theoretical capacity limits and poor cycling stability resulting from structural collapse and dissolution.^[^ ^6^, ^7^, ^8^ ^]^ Shifting the charges stored to conversion chemistry by leveraging the redox reaction of halogens (e.g., I, Br, and Cl) presents a compelling avenue because their rich valance states allow for a large amount of charge transfer and they are abundantly available.^[^ ^9^, ^10^ ^]^

Iodine is an especially appealing choice because of its solid nature at ambient temperature and its favorable redox potential in aqueous electrolytes, which have been intensively investigated in metal‐based iodine batteries such as Li‐I_2_,^[^ ^11^, ^12^ ^]^ Na‐I_2_,^[^ ^13^ ^]^ Zn‐I_2_,^[^ ^14^ ^]^ Mg‐I_2_,^[^ ^15^, ^16^ ^]^ and Al‐I_2_^[^ ^17^, ^18^ ^]^ batteries. Currently, many studies are focusing on mitigating the iodine shuttle effect to enhance the performance of iodine redox electrodes through approaches such as physical/chemical domain limitation,^[^ ^9^, ^10^, ^19^ ^]^ utilization of single‐atom catalysts,^[^ ^20^ ^]^ and development of novel electrolytes to promote their practical applications.^[^ ^21^, ^22^, ^23^ ^]^ However, traditional iodine electrodes typically operate involving a single electron transfer of I^0^/I^−^, yielding a theoretical specific capacity of only 211 mAh g^−1^ at a relatively low voltage of 0.54 V versus SHE in dilute electrolytes,^[^ ^24^, ^25^ ^]^ which also restrict the energy density.

Recent advancements in electrolyte development have enabled the precise facilitation of the I^+^/I^0^/I^−^ two‐electron reactions, concurrently bringing a predictable capacity increase to 422 mAh g^−1^ and elevating the operating voltage to ≈1.07 V versus SHE due to the high redox potential of I^+^/I_2_ couple.^[^ ^26^, ^27^, ^28^, ^29^ ^]^ A representative study by Liang et al. demonstrated that an innovative electrolyte formulation—comprising ZnCl_2_: LiCl: ACN in a 19:19:8 ratio—enables the iodine electrode to achieve a specific capacity of 594 mAh g^−1^ by harnessing the I^+^/I^0^/I^−^ two‐electron reactions, while concurrently maintaining cycling stability over 6000 cycles. The high concentration of ZnCl_2_ is pivotal, as the nucleophilic capability of Cl^−^ is sufficiently robust to form a stable yet redox‐active ICl interhalogen compound.^[^ ^30^ ^]^ The reversible conversion of the I^+^/I_2_ couple is intrinsically linked to the decreased activity of H_2_O in the electrolyte, which inhibits the hydrolysis that typically results from the interaction of soluble polar triiodide (I_3_^−^) species, generated by the reaction between I^−^ and I_2_, with polar H_2_O molecular networks.^[^ ^31^, ^32^, ^33^ ^]^ Furthermore, the formation of charge‐transfer interhalogen complexes, such as ICl or ICl_3_, stabilizes I^+^ through the establishment of strong halogen bonds with the formation of a σ hole.^[^ ^34^ ^]^ On the other hand, the stable interhalogen bond also contributes additional charge transfer by exploiting the redox reaction of Cl^0^/Cl^−^ itself. Zhi et al. proposed a novel three‐electron transfer cell mode utilizing the I^+^/I^−^ and Cl^0^/Cl^−^ conversion reactions in a 30 *m* ZnCl*_2_* electrolyte, achieving an unprecedented energy density of 905 Wh kg^−1^.^[^ ^35^ ^]^ However, the competing oxygen evolution reaction (OER) triggers the Cl_2_ evolution reaction (ClER) due to the powerful parasitic reactions that still occur in the aqueous electrolytes.^[^ ^36^ ^]^ Additionally, the propensity of Cl_2_ to dissolve in water exacerbates the instability of Cl_2_ at the cathode. To further enhance the reversibility of the system, it is essential to adjust the solvation structure of the electrolyte to disrupt the hydrogen bonding network of H_2_O, thereby mitigating its reactivity and reducing OER side reactions.

Along this line, we further investigate viable strategies for manipulating the solvation structure of the electrolyte. In this work, we introduce a new WiDES electrolyte with diverse solvation configurations based on ZnCl_2_, choline chloride (ChCl), EG, and H_2_O for AZIBs. ChCl provides a Cl source while acting as a hydrogen bond donor, which can form a deep eutectic solvent electrolyte with the hydrogen bond acceptor ZnCl_2_, lowering the melting point of the electrolyte to increasing the solubility of ZnCl_2_ thus further decreasing the activity of the water. The polar groups within EG interact with Zn^2+^, facilitating a reconfiguration of the solvated Zn^2^⁺ structure. Spectroscopic characterization and theoretical calculations reveal that EG remodels the hydrogen bonding network by replacing water in the solvated structure, thus reducing the amount of water and leading to water redistribution. Consequently, the incorporation of EG as a co‐solvent expands the oxidation electrochemical stability window (ESW) from 3.65 V to 4.02 V (versus Ag/AgCl), which diminishes the likelihood of the OER, while enhancing the competition between the chlorine‐redox reaction (ClRR) from a CE of 60.9% to 82%, and increasing the first‐cycle CE from 68.2% to 98.7%. The unique cathode‐electrolyte coupling promotes I/Cl immobilization through the formation of I‐Cl interhalogen compounds, facilitating highly reversible three‐electron reactions of I^+^/I^0^/I⁻ and Cl^0^/Cl^⁻^. This innovation allows the iodine cathode to achieve a capacity of 987 mAh g^−1^ at a low current density of 100 mA g^−1^, resulting in an extraordinarily high energy density of 1278 Wh kg^−1^, with a CE sustained above 91.3%. The assembled pouch cell demonstrates a capacity nearing 3.72 mAh cm^−2^ at 0.5 mA cm^−2^ with an energy density of 4.52 mWh cm^−2^, maintaining a CE of 86.7% after 50 cycles, and is capable of illuminating several LED light beads, thereby establishing a foundation for practical applications.

## 2 Results and Discussion

### 2.1 Electrochemical Investigation on the Redox Reversibility

**Figure 1:**
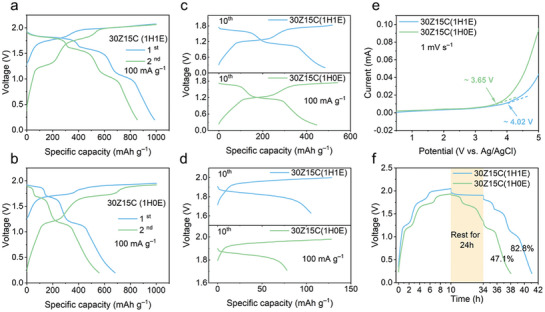
Electrochemical performance of Zn||I_2_ batteries in 30Z15C(1H0E) and 30Z15C(1H1E) electrolytes. The GCD profiles in the a) 30Z15C(1H1E) and b) 30Z15C(1H0E) electrolytes at a current density of 100 mA g^−1^ for the 1st and 2nd cycles, with a charging capacity limit set of 1000 mAh g^−1^. A comparison of the GCD profiles within the voltage range of c) 0.2–1.8 and d) 1.7–2.0 V highlights the reversibility of the IRR and ClRR. e) LSV curves, representing the cathodic stable voltage window of various electrolytes at a scanning rate of 1 mV s^−1^. f) Self‐discharge performance after resting 24h period.

Iodine anchored on a high surface area activated carbon (AC) with a predetermined content of 33 wt.% was used as the working electrode in this study (see Experimental Section, Figures S1 and S2, Supporting Information). To investigate the effect of EG co‐solvent in the WiDES electrolyte on electrochemical performance, we evaluated the galvanostatic charge‐discharge (GCD) profiles of the iodine cathode in two different electrolytes: the EG‐containing and the EG‐absent electrolytes. Specifically, the electrolytes are denoted as 30Z15C(1H1E) (30 *m* ZnCl_2_ + 15 *m* ChCl in a mixed solvent of EG and H_2_O at a 1:1 volume ratio) and 30Z15C(1H0E) (30 *m* ZnCl_2_ + 15 *m* ChCl in H_2_O without EG). As shown in **Figure** **1**a,b, both full Zn||I_2_ cells exhibit three distinct reversibility charge and discharge plateaus, with the two lower voltage plateaus occurring at ≈1.1 and ≈1.6 V, corresponding to the typical iodine‐based redox reaction (IRR), I^−^ ↔ I^0^ and I^0^ ↔ I^+^, while the high voltage plateau at ≈1.85 V is assigned to the Cl^−^redox reaction, that is, Cl^−^/Cl° couple. Unless otherwise specified, voltages in this paper are referenced versus Zn^2+^/Zn. Noticeably, the discharge capacity of the iodine cathode in the 30Z15C(1H1E) electrolyte shows a significant enhancement, achieving a capacity of 987 mAh g^−1^ based on the iodine mass, coupled with a high CE of 98.7% in the first cycle, with a charge capacity limit set to 1000 mAh g^−1^, to which the AC contributed ≈ 150 mAh g^−1^ (Figure S3, Supporting Information). When accounting for the contribution of AC, the cathode still delivers a capacity of ≈357 mAh g^−1^_I2+AC_, given that AC provides minimal capacitive capacity. In contrast, the 30Z15C(1H0E) electrolyte yields only a capacity of 682 mAh g^−1^, accompanied by a poor CE of 68.2%. This pronounced difference underscores the enhanced reversibility of the active redox reactions facilitated by the incorporation of the EG co‐solvent.

To further evaluate and confirm the enhanced capacity contribution, we conducted GCD tests within the voltage ranges of 0.2–1.8 V and 1.7–2.0 V, corresponding to the IRRs and ClRRs, respectively. Interestingly, the reversibility of both reactions improves upon the incorporation of EG. Specifically, the reversibility of the IRR increases from 82.2% to 94.2% (Figure 1c). Additionally, the reversible capacity of the ClRR is improved from 274 to 361 mAh g^−1^ during the first cycle, with the reversible capacity stabilizing at 105 mAh g^−1^ at the 10th cycle (Figure 1d). Concurrently, the CE rises from 60.9% for the 30Z15C(1H0E) electrolyte to 82.0% for the 30Z15C(1H1E) electrolyte. The significantly improved reversibility of both IRR and ClRR in the 30Z15C(1H1E) electrolyte can be attributed to the modification of the solvation structure induced by EG. The EG molecule, characterized by its two O‐H bonds and relatively high polarity, acts as a hydrogen bond donor. In the presence of the strong Lewis acid ZnCl_2_, which serves as a hydrogen bond acceptor, the oxygen atoms in EG can effectively replace the oxygen in the water molecules and form bonds with Zn^2^⁺ through hydrogen bonding. This process disrupts the hydrogen bonding network in H_2_O, thereby reducing its overall activity. As manifested by the linear sweep voltammetry (LSV), the onset potential for the OER in the electrolyte increases from ≈3.65 to 4.02 V versus Ag/AgCl after EG co‐solvated (Figure 1e). Consequently, this shift enables the ClRR to more effectively compete with the OER. Furthermore, the reduced activity of water after the addition of EG significantly enhances the self‐discharge behavior of the Zn||I_2_ battery, improving from 47.1% to 82.8% following a 24h resting period (Figure 1f; Figure S4, Supporting Information). This improvement underscores the restricted formation of soluble polyiodides in the 30Z15C(1H0E) electrolyte.

Of note, a basic concentration of 30 *m* ZnCl_2_ combined with 15 *m* ChCl was chosen as 30Z15C due to the superior reversibility of ClRR observed in this formulation compared to lower concentrations (Figure S5a, Supporting Information), and its significantly lower viscosity relative to a more concentrated solution (Figure S5b, Supporting Information). After confirming the beneficial effect of the EG co‐solvent on reaction reversibly, we further studied the impact of varying amounts of EG on the electrochemical behaviors of Zn||I_2_ batteries. Specifically, deviations in the H_2_O: EG volume ratio resulted in the impaired specific capacities of the Zn||I_2_ batteries (Figure S6, Supporting Information). Within the stable WiDES system, EG and H_2_O serve distinct functions to collectively enhance the ionic conductivity of the composite electrolyte. The strongly polar H_2_O molecules effectively ionize the salt, thereby promoting ionic conductivity. Conversely, the larger, less polar EG molecules impede ion migration; thus, an excess of EG within the electrolyte substantially decreases ionic conductivity (Figure S7, Supporting Information) and reduces discharge capacity. Consequently, we maintained a 1:1 molar ratio of H_2_O to EG for the subsequent WiDES studies.

### 2.2 Solvation Environment Changes

**Figure 2:**
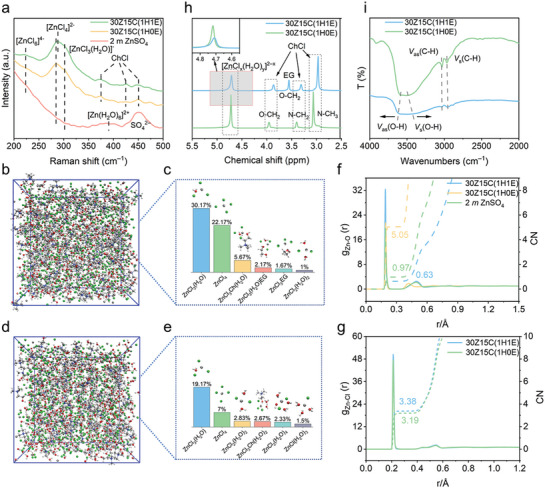
Spectroscopy and MD analysis of the 30Z15C(1H1E) and 30Z15C(1H0E) electrolytes. a) Raman spectra. b) and d) Snapshot of MD simulation boxes, where the gray, green, red, white, light gray, and blue atoms represent Zn, Cl, O, H, C, and N, respectively. c) and e) Dominant clusters and their occurrence, f) and g) RDF g(r) in solid lines and coordination number CN in dotted lines of Zn^2+^ solvation. h) NMR spectra, and i) FTIR spectra.

To trace the origins of these performance discrepancies, the solvation structures and detailed interaction between the two electrolytes were explored in detail via Raman, molecular dynamics (MD) simulation, Nuclear magnetic resonance (NMR), and Fourier transform infrared (FTIR). The Raman spectra of both electrolytes present typical vibrations of Zn─Cl complexes at ≈285 and ≈300 cm^−1^, attributed to the *v*_1_ vibrations of [ZnCl_4_]^2−^ and [ZnCl_3_(H_2_O)]^−^, respectively (**Figure** **2**a).^[^ ^37^, ^38^, ^39^ ^]^ Notably, the addition of co‐solvent EG results in slight redshifts of 4 cm^−1^ of the [ZnCl_4_]^2−^ and a peak emerges at ≈225 cm⁻,^1^ which corresponds to the *v*₁ vibration of [ZnCl_6_]^4^⁻.^[^ ^40^, ^41^, ^42^ ^]^ These indicate an enhanced interaction between Zn^2+^ and Cl⁻, which likely contributes to the observed reduction in ligand water content within the solvated structure,^[^ ^29^ ^]^ which is associated with the decrease in water content and the reorganization of the hydrogen bonding network following EG incorporation. Additionally, new peaks at ≈375, 425, and 450 cm⁻^1^ are attributed to various methyl rocking and bending motions in ChCl (Figure S8, Supporting Information).^[^ ^43^ ^]^ Besides, the dehydration of [Zn(H_2_O)_6_]^2+^ is also evident, driven by the formation of an increased number of Zn─Cl complex ions in the electrolytes, as reflected in the disappearance of the vibration at 390 cm^−1^ in a 2 *m* ZnSO_4_ dilute solution.

MD simulations are further used to decode the solvation structure changes after the EG solvent partially replaces H_2_O in the electrolyte. A snapshot of the simulation frame and cluster distribution for 30Z15C(1H1E) is presented in Figure 2b and Table S1 (Supporting Information), where it is noted that a portion of Zn^2+^ appeared to be coordinated with EG to form structures such as [ZnCl_3_(H_2_O)(EG)]^−^ and [ZnCl_4_(EG)]^2−^ etc., indicating that EG can replace part of the water in the Zn^2+^ solvation structure shell compared to the 30Z15C(1H0E) (Figure 2d). The introduction of EG strips H_2_O molecules from the initial Zn^2+^ solvated structure, thereby transforming Zn^2+^ from water‐rich to water‐poor clusters (Figure 2c,e). For instance, the water‐rich [ZnCl_2_(H_2_O)_4_]^0^ and [ZnCl(H_2_O)_3_]^+^ clusters are no longer the predominant species, and the proportion of [ZnCl_2_(H_2_O)_2_]^0^ decreases from 2.83% to 1% upon the addition of EG. Controversy, the proportions of water‐poor main species, [ZnCl_3_(H_2_O)]^−^ and [ZnCl_4_]^2−^, both show substantial increases, from 19.17% to 30.17% and from 7% to 22.17%, respectively. Further validation is provided by radial distribution functions (RDFs, g(r)) and coordination numbers (CN, n(r)) of Zn─O and Zn─Cl in the two electrolytes (Figure 2f,g). With EG partly replacing H_2_O, the coordination number of Zn─O (H_2_O) is reduced from 0.97 to 0.63, while that of Zn─Cl increases from 3.19 to 3.38, indicating strengthened Zn─Cl association and weakened Zn‐H_2_O hydration. Of note, the coordination Zn‐H_2_O is much lower than the 2 *m* ZnSO_4_ electrolyte of 5.05. Additionally, the coordination numbers for Zn^2+^ and EG, Cl, and EG are only 0.41 and 0.062, respectively (Figure S9, Supporting Information). Since EG hardly coordinates with Cl, the coordination of EG to Zn^2+^ will not affect the coordination of Cl to Zn^2+^.

To further detect the specific intermolecular interactions introduced by the addition of EG, we explored these interactions using NMR ^1^H spectroscopy. As shown in Figure 2h, upon co‐solvating EG into the electrolyte, the ^1^H signals from the ‐OH groups, vested in resonances located at ≈4.72 ppm, become broader and weaker, and undergo a downfield chemical shift.^[^ ^44^, ^45^, ^46^ ^]^ Given that most H_2_O is coordinated within the Zn^2+^ solvation structure, the signal is contributed to the [ZnCl*_x_* (H_2_O)*_y_*]^2‐^ *^x^* complex. The result reflects that EG reduces the hydrogen bonding among H_2_O molecules, which may be caused by the coordination of Zn^2+^≈ O^δ−^ in EG or the hydrogen bond of Cl≈ H^δ+^‐ O^δ−^ in EG.^[^ ^47^, ^48^ ^]^ Besides, compared to the 30Z15C(1H0E) electrolyte, the ^1^H attributed to the methyl and ethyl ‐OH or ‐NH in ChCl show potential hydrogen bonding with O of EG due to their eutectic process,^[^ ^49^ ^]^ as the three peaks at 3.95, 3.4, and 3.06 ppm at different positions in Ch^+^ have also shifted to the lower field with the addition of EG. Additionally, the manipulated hydrogen bond can be revealed by FTIR spectra (Figure 2i). We observe that the symmetric stretching vibration of ‐OH at 3400 cm^−1^ and the asymmetric stretching vibration of ‐OH at 3600 cm^−1^ become broader and weaker after the introduction of EG, which is caused by the strong coordination of the hydroxyl group between Zn^2+^ and EG.^[^ ^50^ ^]^ The above results support that the addition of EG can change the solvation environment of Zn^2+^ to make the reduction of water activity and broaden the onset oxidation potential of the electrolyte, which is the main reason for the reduction of OER, and therefore the CE of ClRR is greatly increased.

### 2.3 Ion Storage Mechanism

**Figure 3:**
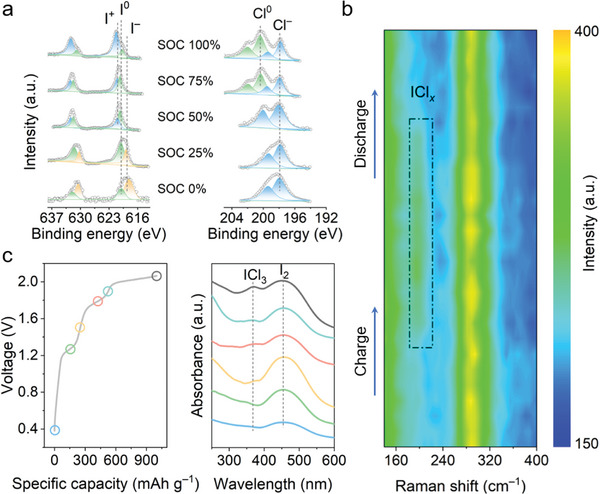
Illustration of the mechanism of Zn||I_2_ batteries in 30Z15C(1H1E) electrolyte. a) XPS spectra of the I_2_/AC cathode at different SOCs (left: I element, right: Cl element), b) In situ Raman spectra of I_2_/AC cathode. c) Ex situ UV–vis spectra of I_2_/AC cathode in 30Z15C(1H1E) electrolyte at different SOCs.

The formation of interhalogen compounds positively impacts the immobilizing of I and Cl by preventing the formation of polyiodides. To investigate the mechanism of ion transformation within the cathode, X‐ray photoelectron spectroscopy (XPS) characterizations of elemental I and Cl at different states of charge (SOC) were observed. As shown in **Figure** **3**a, at a fully charged state (SOC of 100%), the fitted peaks at ≈621 and ≈632.5 eV are attributed to the oxidized state of I, that is, I^+^, while peaks at ≈620 and ≈631 eV correspond to the conform to I‐I bonds derived from internal free I_2_ molecules, that is, I^0^.^[^ ^51^ ^]^ Upon discharge, when the SOC is at 75%, the fitted content of I^+^ decreases from 71.7% to 53.7%, while I^0^ increases from 28.3% to 46.3% (Table S2, Supporting Information). At a SOC of 25%, I^+^ is completely reduced to I^0^, and I^0^ is gradually reduced to I^−^ at ≈618 and ≈630.2 eV. Notably, the energy band of I shifts by ≈0.3 eV compared to the SOC 100%, representing electron redistribution due to bonding with chlorine species during charging/discharging.^[^ ^35^ ^]^ Similarly, peaks attributed to Cl^−^ located at ≈197.9 eV and the peaks attributed to Cl^0^ located at ≈199 eV can be observed in a fully charged state. At a SOC of 75%, the fitted content of Cl^0^ decreased from 64% to 54%, while Cl^−^ increased from 36% to 46%. At 50% SOC, only the peaks attributed to Cl^−^ are observed. Notably, the Cl^−^ peak is still detectable upon further discharge. To further confirm this persistence, we performed XPS characterization of elemental chlorine in both the pristine state and the fully discharged state (Figure S10, Supporting Information), confirming the continual presence of Cl^−^ at the cathode surface after cycling due to the immobilization of iodine. Additionally, Zn content was detected in the I_2_/AC cathode at the voltage range of 1.1–0.2 V, leading us to hypothesize the formation of ZnI_2_ or Zn(I_3_)_2_ (Figure S11, Supporting Information).

Ex situ high‐angle annular dark field scanning transmission electron microscope (HAADF‐STEM) images and corresponding energy dispersive X‐ray spectroscopy (EDX) elemental mappings of Cl, I, and Zn elements are used to evaluate the elemental distribution as well as the relative content at different SOCs (Figure S12; Table S3, Supporting Information). After the first cycle, the elements I, and Cl are uniformly distributed at the cathode. The gradual increase in the relative content of Cl and decrease in the relative content of I with depth of SOC corresponds to an increase in the iodine valence state and the generation of polychlorinated iodine, which is consistent with the XPS results. A surge in the relative content of Zn has been detected at 0% SOC, which may correspond to the generation of the ZnI*_x_* compound. However, it is noteworthy that almost no Zn is present at 100% SOC, suggesting that the product of the complete oxidation of the I_2_/AC cathode is not ZnICl*_x_*.

To precisely confirm the anionic evolution during charge and discharge, in situ Raman spectra were further performed on the cathode. Figure 3b shows that except for the strong peaks at ≈290 to ≈305 cm^−1^ owing to the [ZnCl_3_(H_2_O)]^−^ and [ZnCl_4_]^2−^ species existed in the electrolyte, a new peak appears at ≈201 cm^−1^ when charging to 1.4 V, and it progressively intensifies with further charging. When discharging to 1.2 V, contrastively, the peak gradually disappeared. We attribute the new peak to the combination product of I^+^ and Cl^−^, that is, an ICl interhalogen.^[^ ^52^ ^]^ As the charging process continues, ICl will keep oxidizing to ICl_2_^−^ or ICl_3_^−^.^[^ ^35^, ^53^ ^]^ Notably, theoretical product energy barriers derived from density functional theory (DFT) calculations show that ICl will first bind to Cl^−^ with 2.15 eV of energy released to generate ICl_2_^−^, which then absorbs 4.33 eV of energy to be oxidized to ICl_2_, and finally binds to Cl^−^ with 2.1 eV of energy released to generate ICl_3_^−^, instead of the direct oxidation of Cl^−^ to the chlorine gas being released (Figure S13, Supporting Information). In addition, ex situ ultraviolet‐visible (UV–vis) spectroscopy spectra at different SOCs were tested to further demonstrate the reaction process (Figure 3c). The main peak at ≈458 nm, representing the iodine monomer, reaches its highest intensity at 25% SOC, indicating a conversion from I^−^ to I^0^. At 50% SOC, a new peak at ≈360 nm, which can be assigned to the ICl_3_^−^ species appears and intensifies after fully charged, in correspondence with the in situ Raman.^[^ ^54^ ^]^ Note that the peak of triiodide (I_3_^−^) is not detected here but the peak of I^−^, therefore, the product is determined as ZnI_2_ in the 0.2 V rather than the Zn(I_3_)_2_. The conversion of I^−^ to I^0^ as well as I^0^ to I^+^ can still be detected by XPS of the next cyclic charging state, suggesting that the cathode product is well reversible (Figure S14, Supporting Information).

### 2.4 Electrochemical Energy Storage Performance

**Figure 4:**
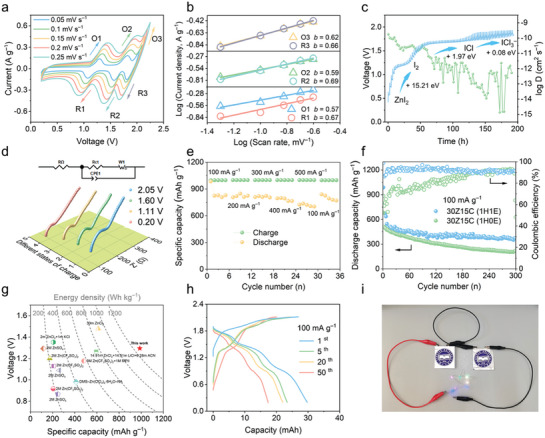
Electrochemical Energy Storage Performance. a) CV curves with different sweep rates from 0.05 to 0.25 mV s^−1^. b) The plots of the oxidation and reduction peak‐current with the function of the root of sweep rates. c) GITT curves, diffusion coefficients calculated from GITT potential curves, and energy barriers for product transformation. d) EIS at different SOCs and fitted circuit diagrams corresponding to the EIS. e) Rate performance of the I_2_/AC electrode in 30Z15C(1H1E) electrolytes. f) Cycling performance of the I_2_/AC electrode in 30Z15C(1H1E) and 30Z15C(1H0E) electrolytes. g) Performance comparison of Zn‐I_2_ batteries. h) GCD profiles of Zn‐I_2_ pouch cell at a 0.5 mA cm^−2^ current density. i) Digital image of Zn‐I_2_ pouch cell.

By leveraging the co‐solvated effect of EG and interhalogen working mechanism, we further assess the dynamic and stability properties of the iodine cathode in the 30Z15C(1H1E) electrolyte. The cyclic voltammetry (CV) curves of the Zn||I_2_ cell in the system, recorded at various sweep rates, are shown in **Figure** **4**a. Kinetic analysis was conducted using the relationship between the logarithms of current (i) and sweep rate (ν), depicted in the log *i*‐log *ν* point plots (Figure 4b), and employing the power law equation *i = av^b^*, where the parameter *b* can be indicative of non‐diffusion‐controlled behavior (*b* = 1.0) or diffusion‐controlled behavior (*b* = 0.5).^[^ ^55^ ^]^ The *b* values of the three pairs of redox peaks corresponding to the I^0^/I^−^, I^+^/I^0^, and Cl^0^/Cl^−^ are all close to 0.5, signifying diffusion‐controlled processes. The galvanostatic intermittent titration technique (GITT) was subsequently utilized to elucidate ionic diffusion phenomena. As shown in Figure 4c, the diffusion coefficient during charging is maintained at 10^−9^∼10^−15^ cm^−2^ s^−1^, wherein the diffusion coefficient decreases by 1, 2, and 2 orders of magnitude, as charged to ∼1.2, 1.5, and 1.7 V, respectively. The reduction is attributable to the transition from ZnI_2_ to I_2_, subsequent transformation to ICl, and ultimately to ICl_3_^−^, where bond formation and dissociation involve substantial energy barriers, corresponding to +15.21, +1.97, and +0.08 eV, respectively, thus resulting in slow kinetics.^[^ ^56^ ^]^ Notably, the potentials are much lower than that observed in the GCD profiles and CV curves due to a reduced current charge rate of 50 mA g^−1^ and a prolonged resting period of 2 h. In addition, electrochemical impedance spectroscopy (EIS) measurements across different SOCs were conducted to evaluate the resistance of the battery as well as the ionic diffusion coefficient (Figure 4d). Upon charging, the internal resistance (R_Ω_) increases from 83.3 to 140 Ω, and the charge transfer resistance (R_ct_) increases from 68.2 to 120.9 Ω, both showing an upward trend (Table S4, Supporting Information), potentially attributed to gradually newly formed ICl*_x_* product during the charging retard multiple ion/electron transfer steps along with the accumulation of chloride ions.^[^ ^57^, ^58^ ^]^ The ion diffusion coefficient fitted based on EIS data is −13 and −12 and shows a gradually decreasing trend, which is in coincidence with GITT. The rate performance of the Zn||I_2_ cell was carried out in 30Z15C(1H1E) electrolytes at current densities of 100, 200, 300, 400, and 500 mA g^−1^, which exhibits high reversible capacities of 828, 820, 810, 793, and 721 mAh g^−1^ at a limited charging capacity of 1000 mAh g^−1^, respectively (Figure 4e; Figure S15, Supporting Information).

We evaluated the cycling performance of the Zn||I_2_ cells with and without EG co‐solvated at a low current density of 100 mA g^−1^ (Figure 4f). Although the capacity decayed rapidly during the initial cycles, possibly attributable to partial iodine cathode dissolution, it stabilized by the 10th cycle at ≈450 mAh g^−1^, maintaining ≈300 mAh g^−1^ over 300 cycles (over ≈100 days), with a CE consistently ≈ 94%. In contrast, in the 30Z15C(1H0E) electrolyte without EG substitution, the battery presents an initial CE of only 65% and a capacity of only 200 mAh g^−1^ after 300 cycles (Figure S16, Supporting Information). The long cycle was performed at a current density of 300 mA g^−1^ and the capacity was maintained at 220 mAh g^−1^ after 1000 cycles with a CE of 96.3% (Figure S17, Supporting Information). The calculated first cycle energy density of the Zn||I_2_ battery is 1278 Wh kg^−1^, which is superior to most of the relevant aqueous electrochemical energy storage systems (Figure 4g; Table S5, Supporting Information).^[^ ^24^, ^28^, ^30^, ^35^, ^59^, ^60^, ^61^, ^62^, ^63^, ^64^, ^65^ ^]^ It is worth noting that the above calculations of specific capacity and energy density are based on the mass of the cathode active materials, whereas the pouch cell is calculated based on the cathode (I_2_+AC) and the anode (Zn). To exhibit the performance of Zn‐I_2_ cells in practical applications, we assembled pouch cells in 30Z15C(1H1E) electrolytes (Figure 4h). Pouch cells have an open‐circuit potential of 1.22 V, exhibiting 3.72 mAh cm^−2^ reversible area capacity at 0.5 mA cm^−2^ current densities, and can be stably cycled 50 times. To evaluate the effectiveness of the pouch cell, it was used as a power source for a small light bulb (Figure 4i).

## 3 Conclusion

In this study, we successfully developed a novel WiDES electrolyte comprising 30 *m* ZnCl_2_ and 15 *m* ChCl in an EG/water solvent for high‐utilization aqueous zinc‐iodine batteries. The partial substitution of water with EG in the electrolyte plays a crucial role in enhancing its electrochemical properties. This substitution significantly alters the solvation structure of Zn^2+^, disrupts the hydrogen bonding network of H_2_O, and increases the electrochemical stability window from 3.65 to 4.02 V. This modification enables high reversibility of the I^−^/I^0^/I^+^ and Cl^−^/Cl^0^ redox reactions, contributing to a substantial improvement in battery performance. The aqueous Zn‐I_2_ full cell delivers an ultra‐high specific capacity of 987 mAh g^−1^_I2_ and an energy density of 1278 Wh kg^−1^, enhanced by ≈300 mAh g^−1^ in contrast to the EG‐absent electrolyte. The cell's Coulombic efficiency remains above 91.3% after 300 cycles, demonstrating excellent cycling stability. Ex situ and in situ analyses, including XPS, Raman, and UV–vis spectroscopy, elucidate the changes in valence states, particle contents, and ion transformation mechanisms at different SOCs. This work provides a new perspective on designing WiDES electrolytes for zinc‐based halogen batteries. The demonstrated improvements in capacity, energy density, and cycle life underscore the potential of EG co‐solvents to significantly advance the performance of aqueous zinc‐iodine batteries, paving the way for future high‐voltage and high‐capacity energy storage solutions.

## 4 Experimental Section

### Preparation of EG/Water Hybrid DES Electrolytes

To prepare the aqueous deep eutectic solvent (DES) electrolyte, 30 mmol of zinc chloride (ZnCl_2_, anhydrous, ≥98%, Sigma Aldrich) and 15 mmol of choline chloride (C_5_H_14_ClNO, >98.0%, TCI) were added to 1 g of pure water, then heated and stirred at 70 °C until clarified and clear (noted as 30Z15C(1H0E)). In addition, DES electrolytes of 15Z7.5C(1H0E), 40Z20C(1H0E), were prepared as described above. Then, a portion of the water was substituted with ethylene glycol (EG, >99%, Aladdin) to make the water: ethylene glycol mass ratios of 3:1, 2:1, 1:1, 1:2, 1:3, respectively (denoted as 30Z15C (3H1E), 30Z15C(2H1E), 30Z15C(1H1E), 30Z15C(1H2E), 30Z15C(1H3E)).

### Preparation of I_2_/AC Cathodes

First, 100 mg of AC (Ultra large specific surface area disordered porous carbon, specific surface area >3000 m^2^ g^−1^, XFNANO) and 100 mg of iodine monomers were added to 30 mL of pure water and ultrasonicated 3 times for 1 h each time to induce iodine adsorption into AC. Centrifuge and dry at 60 °C for 12 h. I_2_/AC was mixed with Ketjen black (KB, XFNANO) and Polytetrafluoroethylene (PTFE, Alfa Aesar) with a mass ratio of 8:1:1 in the mortar for 15 min and then pressed into free‐standing films on a roller machine, followed by drying in an oven at 60 °C for 8 h. Ultimately, the content of the active substance iodine accounted for 30–35 wt.% of the free‐standing films.

### Materials Characterizations

The X‐ray diffraction (XRD) pattern was obtained on a Rigaku D/Max‐2500/PC X‐ray diffractometer with a scan range of 5–90° at a scan rate of 5° min^−1^. The thermogravimetric analysis was performed on DTG‐60A from room temperature to 450 °C with a heating rate of 5 °C min^−1^ in air atmosphere. The viscosity of the electrolytes was examined by Brookfield DV‐2 pro. Nuclear Magnetic Resonance (NMR) spectroscopy was carried out on a Bruker 400 M. Fourier transform infrared spectroscopy (FTIR) was carried out on a Frontier FT‐IR. The Raman spectra of electrodes and electrolytes were obtained on a Renishaw with a laser excitation wavelength of 532 nm. The investigation of the valence states of the elements was conducted on an X‐ray photoelectron spectrometer (XPS, Nexsa) with monochromatic Al Kα radiation. High‐angle annular dark field‐scanning transmission electron microscopy (HAADF‐STEM) images and energy dispersive X‐ray spectroscopy (EDX) elemental mappings were obtained on JEM‐F200. Ultraviolet‐visible spectra were obtained on a UV‐2550 (Shimadzu, Kyoto, Japan).

### Theoretical Calculations

MD simulations were performed on 30Z15C(1H1E), 30Z15C(1H0E), and 2 m ZnSO_4_ solutions. The MD simulations used GAFF2 force field parameters for all the ions.^[^ ^66^, ^67^, ^68^ ^]^ Other small molecules were optimized via Gaussian 16 software with a level of B3LYP/def2tzvp. Moreover, the RESP2 charge5 was used to obtain more accurate results. The initial structures were modeled via Packing Optimization for Molecular Dynamics Simulations (Packmol) program6 based on the periodic box of 5*5*5 nm^3^. All MD simulations were performed using Gromacs software (2019.5 version) according to previous simulation processes. In detail, the 1500‐step steepest descent method and 1500‐step conjugate gradient method were used to avoid unreasonable contact with the system. NPT ensemble was used to pre‐equilibrate the system, and V‐rescale temperature coupling and Parrinello‐Rahman pressure coupling were used to control the temperature to 298 K. The pressure was maintained at 1 atm, the non‐bonding cutoff radius was 1.2 nm, and the integration step was 1 fs. The systems were then cooled from 298 to 150 K for 1 ns, and maintained at 150 K for 1 ns, and subsequently annealed from 150 to 298 K in 1 ns. After that, the simulation boxes were equilibrated at 298 K in an NPT ensemble for another 1 ns. A 20 ns production run in an NVT ensemble under Nose‐Hoover thermostat with a time constant of 1 ps at 298 K was finally conducted. Only the final 5 ns were sampled for radial distribution function (RDF) and coordination structure counting analyses. All the initial geometries for DFT calculation were obtained from the gaussview software. The optimization of these geometries were performed via gaussian 16 software with a level of b3lyp/6‐311g(d,p) em = gd3bj, and the vibration analysis was also performed at the same level to ensure no virtual frequency in all obtained geometries. The single point energy calculation of were calculated at a level of b3lyp/def2tzvp em = gd3bj.

### Electrochemical Measurements

All electrochemical measurements on the half‐cells were performed in swagelok cells. For half‐cell assembly, I_2_/AC (≈ 5 mg) was used as the working electrode, Zn (0.1 mm thick, purity) was used as the counter electrode, and glass fiber membrane (Pall Corporation) with a thickness of 330 µm and pore size of 1 µm was used as the separator. For the assembly of pouch cells, freestanding was pressed with a roller machine to Ti mesh (80‐100 mesh) as the cathode (20 mm × 40 mm), Zn foil (0.05 mm) as the anode (23 mm × 43 mm), and glass fiber membrane (Pall Corporation) was used as the separator. All cells were assembled in an air atmosphere. The galvanostatic charge and discharge (GCD) and the galvanostatic intermittent titration technique (GITT) measurements were performed on a Landt battery testing system (CT3001A). The GITT measurements of the battery were performed at a current rate of 50 mA g^−1^ for 20 min with an open‐circuit resting of 2 h. The equation for the diffusion coefficient is shown in Equation (1):

(1) $$D=4/9\pi {\left(\Delta E_{S}/\Delta E_{t}\right)}^{2}r_{s}^{2}/t$$

where Δ*E_S_* and Δ*E_t_* are the potential change of quasi‐equilibrium potential and the potential variation during the current pulse, respectively, *r_s_* is the particles of electrodes, and *t* is the duration time of the pulse. The Cyclic voltammetry (CV) curves and electrochemical impedance spectroscopy (EIS) studies were conducted by an electrochemical workstation (Bio‐logic, SP‐150). The EIS curves were obtained using a voltage amplitude of 10 mV and a frequency range of 10^5^ to 10^−2^ Hz. The diffusion coefficient equation fitted by EIS is shown in Equation (2):

(2) $$D=\;0.5{\left(\mathrm{RT}/n^{2}F^{2}\mathrm{AC}A_{w}\right)}^{2}$$

where *A_w_* can be obtained from Z′  =  R_s_+R_ct_+A_w_ω^−1/2^ in the low‐frequency region, R is the gas constant, T is the absolute temperature, n is the number of transferred electrons, F is Faraday's constant, A is the area of the electrode immersed in the solution, and C is the concentration of the active substance in the electrode. All electrochemical tests were conducted at 25 °C.

## Conflict of Interest

The authors declare no conflict of interest.

## Supporting information



Supporting Information

## Data Availability

The data that support the findings of this study are available from the corresponding author upon reasonable request.

## References

[1] L. Zhang , I. A. Rodriguez‐Perez , H. Jiang , C. Zhang , D. P. Leonard , Q. B. Guo , W. F. Wang , S. M. Han , L. M. Wang , X. L. Ji , Adv. Funct. Mater. 2019, 29, 1902653.

[2] W. Wang , R. Li , Z. Duan , J. Zhao , Y. Qi , Q. Guo , Q. Peng , D. Wang , S. Han , L. Zhang , Chem. Eng. J. 2023, 456, 141019.

[3] L. She , H. Cheng , Z. Yuan , Z. Shen , Q. Wu , W. Zhong , S. Zhang , B. Zhang , C. Liu , M. Zhang , H. Pan , Y. Lu , Adv. Sci. 2024, 11, 2305061. 10.1002/advs.202305061 PMC10953720 37939285

[4] J. Li , Z. Liu , S. Han , P. Zhou , B. Lu , J. Zhou , Z. Zeng , Z. Chen , J. Zhou , Nano‐Micro Lett. 2023, 15, 237. 10.1007/s40820-023-01206-2 PMC10603014 37882885

[5] X. Li , Z. Chen , P. Ruan , X. Hu , X. Yuan , B. Lu , L. Qin , J. Zhou , Nanoscale. 2024, 16, 18835. 39246051 10.1039/d4nr02222j

[6] H. Dong , J. Li , J. Guo , F. Lai , F. Zhao , Y. Jiao , D. J. L. Brett , T. Liu , G. He , I. P. Parkin , Adv. Mater. 2021, 33, 2007548. 10.1002/adma.202007548 33797810

[7] L. Ma , M. A. Schroeder , O. Borodin , T. P. Pollard , M. S. Ding , C. Wang , K. Xu , Nat. Energy. 2020, 5, 743.

[8] X. Li , C. Ji , J. Shen , J. Feng , H. Mi , Y. Xu , F. Guo , X. Yan , Adv. Sci. 2023, 10, 2205794. 10.1002/advs.202205794 PMC10015855 36670056

[9] L. Chai , X. Wang , Y. Hu , X. Li , S. Huang , J. Pan , J. Qian , X. Sun , Adv. Sci. 2022, 9, 2105063. 10.1002/advs.202105063 PMC9685461 36181364

[10] L. Zhang , M. Zhang , H. Guo , Z. Tian , L. Ge , G. He , J. Huang , J. Wang , T. Liu , I. P. Parkin , F. Lai , Adv. Sci. 2022, 9, 2105598. 10.1002/advs.202105598 PMC9069388 35253402

[11] Q. Zhao , Y. Lu , Z. Zhu , Z. Tao , J. Chen , Nano Lett. 2015, 15, 5982. 26241461 10.1021/acs.nanolett.5b02116

[12] Q. Chen , S. Chen , J. Ma , S. Ding , J. Zhang , Adv. Funct. Mater. 2024, 34, 2308272.

[13] T. Zhang , F. Wei , Y. Wu , W. Li , L. Huang , J. Fu , C. Jing , J. Cheng , S. Liu , Adv. Sci. 2023, 10, 2301918. 10.1002/advs.202301918 PMC10323648 37098637

[14] K. Zhang , Z. Jin , Energy Storage Mater. 2022, 45, 332.

[15] H. Tian , T. Gao , X. Li , X. Wang , C. Luo , X. Fan , C. Yang , L. Suo , Z. Ma , W. Han , C. Wang , Nat. Commun. 2017, 8, 14083. 28071666 10.1038/ncomms14083 PMC5234091

[16] I. Huang , Y. Zhang , H. M. Arafa , S. Li , A. Vazquez‐Guardado , W. Ouyang , F. Liu , S. Madhvapathy , J. W. Song , A. Tzavelis , J. Trueb , Y. Choi , W. J. Jeang , V. Forsberg , E. Higbee‐Dempsey , N. Ghoreishi‐Haack , I. Stepien , K. Bailey , S. Han , Z. J. Zhang , C. Good , Y. Huang , A. J. Bandodkar , J. A. Rogers , Energy Environ. Sci. 2022, 15, 4095.

[17] H. Tian , S. Zhang , Z. Meng , W. He , W.‐Q. Han , ACS Energy Lett. 2017, 2, 1170.

[18] S. Zhang , X. Tan , Z. Meng , H. Tian , F. Xu , W.‐Q. Han , J. Mater. Chem. A. 2018, 6, 9984.

[19] X. Guo , H. Xu , Y. Tang , Z. Yang , F. Dou , W. Li , Q. Li , H. Pang , Adv. Mater. 2024, 36, 2408317. 10.1002/adma.202408317 39081106

[20] L. Ma , G. Zhu , Z. Wang , A. Zhu , K. Wu , B. Peng , J. Xu , D. Wang , Z. Jin , Nano Lett. 2023, 23, 5272. 37260235 10.1021/acs.nanolett.3c01310

[21] K. Zhang , Q. Yu , J. Sun , Z. Tie , Z. Jin , Adv. Mater. 2024, 36, 2309838. 10.1002/adma.202309838 37949441

[22] S. J. Zhang , J. Hao , H. Wu , Q. Chen , C. Ye , S. Z. Qiao , Adv. Mater. 2024, 36, 2404011. 10.1002/adma.202404011 38970531

[23] K. Zhang , Y. Ge , Q. Yu , P. Zhang , Y. Feng , Z. Tie , J. Ma , Z. Jin , Energy Storage Mater. 2024, 67, 103296.

[24] X. Li , M. Li , Z. Huang , G. Liang , Z. Chen , Q. Yang , Q. Huang , C. Zhi , Energy Environ. Sci. 2021, 14, 407.

[25] S. Yang , X. Guo , H. Lv , C. Han , A. Chen , Z. Tang , X. Li , C. Zhi , H. Li , ACS Nano. 2022, 16, 13554. 36001394 10.1021/acsnano.2c06220

[26] J. Wang , H. Xu , R. Zhang , G. Sun , H. Dou , X. Zhang , Nanoscale. 2024, 16, 6596. 38466180 10.1039/d3nr06195g

[27] X. Li , S. Wang , D. Zhang , P. Li , Z. Chen , A. Chen , Z. Huang , G. Liang , A. L. Rogach , C. Zhi , Adv. Mater. 2024, 36, 2304557. 10.1002/adma.202304557 37587645

[28] W. Li , H. Xu , H. Zhang , F. Wei , T. Zhang , Y. Wu , L. Huang , J. Fu , C. Jing , J. Cheng , S. Liu , Energy Environ. Sci. 2023, 16, 4502.

[29] L. Miao , R. Wang , W. Xin , L. Zhang , Y. Geng , H. Peng , Z. Yan , D. Jiang , Z. Qian , Z. Zhu , Energy Storage Mater. 2022, 49, 445.

[30] Y. P. Zou , T. T. Liu , Q. J. Du , Y. Y. Li , H. B. Yi , X. Zhou , Z. X. Li , L. J. Gao , L. Zhang , X. Liang , Nat. Commun. 2021, 12, 170. 33419999 10.1038/s41467-020-20331-9 PMC7794333

[31] C. Meng , F. Zhou , H. Liu , Y. Zhu , Q. Fu , Z.‐S. Wu , ACS Energy Lett. 2022, 7, 1706.

[32] H. B. Wang , X. Liu , J. S. Zhong , L. Y. Du , S. Yun , X. L. Zhang , Y. F. Gao , L. T. Kang , Small. 2024, 20, 2306947. 10.1002/smll.202306947 37972273

[33] J. Hao , L. Yuan , Y. Zhu , X. Bai , C. Ye , Y. Jiao , S.‐Z. Qiao , Angew. Chem., Int. Ed. 2023, 62, e202310284. 10.1002/anie.202310284 37548518

[34] G. Cavallo , P. Metrangolo , R. Milani , T. Pilati , A. Priimagi , G. Resnati , G. Terraneo , Chem. Rev. 2016, 116, 2478. 26812185 10.1021/acs.chemrev.5b00484 PMC4768247

[35] G. Liang , B. Liang , A. Chen , J. Zhu , Q. Li , Z. Huang , X. Li , Y. Wang , X. Wang , B. Xiong , X. Jin , S. Bai , J. Fan , C. Zhi , Nat. Commun. 2023, 14, 1856. 37012263 10.1038/s41467-023-37565-y PMC10070632

[36] Q. Zhang , Y. Ma , Y. Lu , Y. Ni , L. Lin , Z. Hao , Z. Yan , Q. Zhao , J. Chen , J. Am. Chem. Soc. 2022, 144, 18435. 36170558 10.1021/jacs.2c06927

[37] D. E. Irish , T. F. Young , J. Chem. Phys. 1965, 43, 1765.

[38] C. Zhang , J. Holoubek , X. Wu , A. Daniyar , L. Zhu , C. Chen , D. P. Leonard , I. A. Rodríguez‐Pérez , J.‐X. Jiang , C. Fang , X. Ji , Chem. Commun. 2018, 54, 14097. 10.1039/c8cc07730d 30488907

[39] Y. Song , Y. Liu , D. Long , X. Tao , S. Luo , Y. Yang , H. Chen , M. Wang , S. Chen , Z. Wei , Adv. Funct. Mater. 2024, 2410305.

[40] J. D. Mackenzie , W. K. Murphy , J. Chem. Phys. 1960, 33, 366.

[41] C. Zhang , W. Shin , L. Zhu , C. Chen , J. C. Neuefeind , Y. Xu , S. I. Allec , C. Liu , Z. Wei , A. Daniyar , J. X. Jiang , C. Fang , P. Alex Greaney , X. Ji , Carbon Energy. 2020, 3, 339.

[42] W. Wang , L. Zhang , Z. Duan , R. Li , J. Zhao , L. Tang , Y. Sui , Y. Qi , S. Han , C. Fang , D. Wang , X. Ji , Carbon Energy. 2024, e577, 10.1002/cey2.577.

[43] K. i. Kim , Q. Guo , L. Tang , L. Zhu , C. Pan , C. h. Chang , J. Razink , M. M. Lerner , C. Fang , X. Ji , Angew. Chem., Int. Ed. 2020, 59, 19924. 10.1002/anie.202009172 32710468

[44] Y. Qiao , C. M. Pedersen , Y. Wang , X. Hou , ACS Sustain. Chem. Eng. 2014, 2, 2576.

[45] T. Zhao , F. Sha , J. Xiao , Q. Xu , X. Xie , J. Zhang , X. Wei , Fluid Phase Equilib. 2015, 405, 7.

[46] A. Wahl , N. Azaroual , M. Imbenotte , D. Mathieu , G. Forzy , B. Cartigny , G. Vermeersch , M. Lhermitte , Toxicology. 1998, 128, 73. 9704907 10.1016/s0300-483x(98)00055-9

[47] L. Geng , J. Meng , X. Wang , C. Han , K. Han , Z. Xiao , M. Huang , P. Xu , L. Zhang , L. Zhou , L. Mai , Angew. Chem., Int. Ed. 2022, 61, e202206717. 10.1002/anie.202206717 35610667

[48] Y. Sui , M. Lei , M. Yu , A. Scida , S. K. Sandstrom , W. Stickle , T. D. O'Larey , D.‐e. Jiang , X. Ji , ACS Energy Lett. 2023, 8, 988. 10.1002/adma.202302595 37604112

[49] K. Jafari , M. Hossein Fatemi , L. Lugo , J. Mol. Liq. 2022, 360, 119521.

[50] A. N. Egorochkin , S. E. Skobeleva , Russ. Chem. Rev. 1979, 48, 1198.

[51] C. Guo , Y. Cao , Y. Gao , C. Zhi , Y.‐X. Wang , Y. Luo , X.‐J. Yang , X. Luo , Adv. Funct. Mater. 2024, 34, 2314189.

[52] W. B. Person , G. R. Anderson , J. N. Fordemwalt , H. Stammreich , R. Forneris , J. Chem. Phys. 1961, 35, 908.

[53] P. Li , X. Li , Y. Guo , A. Chen , R. Zhang , Y. Hou , Q. Xiong , Y. Wang , Z. Chen , J. Zhu , M. Zhu , C. Zhi , Chem. 2024, 10, 352.

[54] M. Torabbeigi , T. Madrakian , A. Afkhami , J. Inclusion Phenom. Macrocyclic Chem. 2009, 67, 127.

[55] H. Lindström , S. Södergren , A. Solbrand , H. Rensmo , J. Hjelm , A. Hagfeldt , S.‐E. Lindquist , J. Phys. Chem. B. 1997, 101, 7717.

[56] Q. Guo , K.‐I. Kim , S. Li , A. M. Scida , P. Yu , S. K. Sandstrom , L. Zhang , S. Sun , H. Jiang , Q. Ni , D. Yu , M. M. Lerner , H. Xia , X. Ji , ACS Energy Lett. 2021, 6, 459.

[57] A. Guerrero , J. Bisquert , G. Garcia‐Belmonte , Chem. Rev. 2021, 121, 14430. 34845904 10.1021/acs.chemrev.1c00214

[58] F. Li , Z. Liu , C. Liao , X. Xu , M. Zhu , J. Liu , ACS Energy Lett. 2023, 8, 4903.

[59] W. Liu , P. Liu , Y. Lyu , J. Wen , R. Hao , J. Zheng , K. Liu , Y.‐J. Li , S. Wang , ACS Appl. Mater. Interfaces. 2022, 14, 8955. 35147408 10.1021/acsami.1c21026

[60] W. Li , K. Wang , K. Jiang , J. Mater. Chem. A. 2020, 8, 3785.

[61] J.‐L. Yang , H.‐H. Liu , X.‐X. Zhao , X.‐Y. Zhang , K.‐Y. Zhang , M.‐Y. Ma , Z.‐Y. Gu , J.‐M. Cao , X.‐L. Wu , J. Am. Chem. Soc. 2024, 146, 6628. 38359144 10.1021/jacs.3c12638

[62] K. K. Sonigara , J. V. Vaghasiya , M. Pumera , Adv. Energy Mater. 2024, 2401321, 10.1002/aenm.202401321.

[63] M. Wang , J. Ma , H. Zhang , L. Fu , X. Li , K. Lu , Small. 2024, 20, 2307021. 10.1002/smll.202307021 37940629

[64] Q. Guo , H. Wang , X. Sun , Y. n. Yang , N. Chen , L. Qu , ACS Mater. Lett. 2022, 4, 1872.

[65] W. Du , L. Miao , Z. Song , X. Zheng , C. Hu , Y. Lv , L. Gan , M. Liu , Chem. Eng. J. 2024, 484, 149535.

[66] R. Galvelis , S. Doerr , J. M. Damas , M. J. Harvey , G. De Fabritiis , J. Chem. Inf. Model. 2019, 59, 3485. 31322877 10.1021/acs.jcim.9b00439

[67] M. Schauperl , P. S. Nerenberg , H. Jang , L.‐P. Wang , C. I. Bayly , D. L. Mobley , M. K. Gilson , Commun. Chem. 2020, 3, 44. 34136662 10.1038/s42004-020-0291-4 PMC8204736

[68] L. Martínez , R. Andrade , E. G. Birgin , J. M. Martínez , J. Comput. Chem. 2009, 30, 2157. 19229944 10.1002/jcc.21224

