# Supplementary material for: Iodine/Chlorine Multi‐Electron Conversion Realizes High Energy Density Zinc‐Iodine Batteries
Source: Adv Sci (Weinh). 2024 Nov 5;12(1):2410988. doi: 10.1002/advs.202410988 (PMC11714215; doi:10.1002/advs.202410988)
Supplement: Supplementary file 1 — Supporting Information [file ADVS-12-2410988-s001.docx]

Supporting Information

**Iodine/chlorine Multi-electron Conversion Realizes High Energy Density Zinc-Iodine Batteries**

Jiajin Zhao, Yan Chen, Mengyan Zhang, Ziqi An, Binbin Nian, Wenfeng Wang, Hao Wu, Shumin Han, Yuan Li* and Lu Zhang*


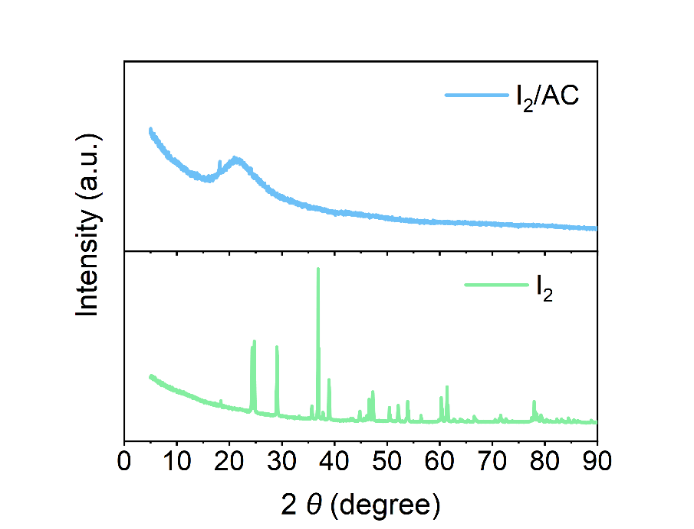


**Figure S1.** XRD patterns of I_2_/AC composite (top) and I_2_ monomers (bottom).


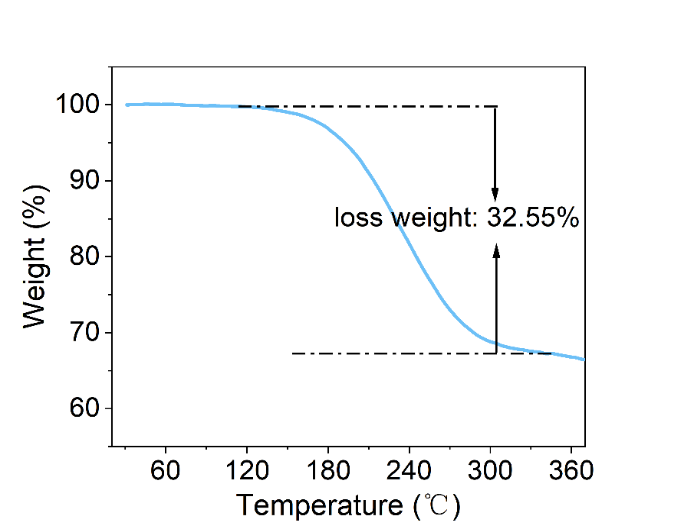


**Figure S2.** TGA curve of the I_2_/AC composite from room temperature to 400℃ at a ramp rate of 5℃ min^–1^.


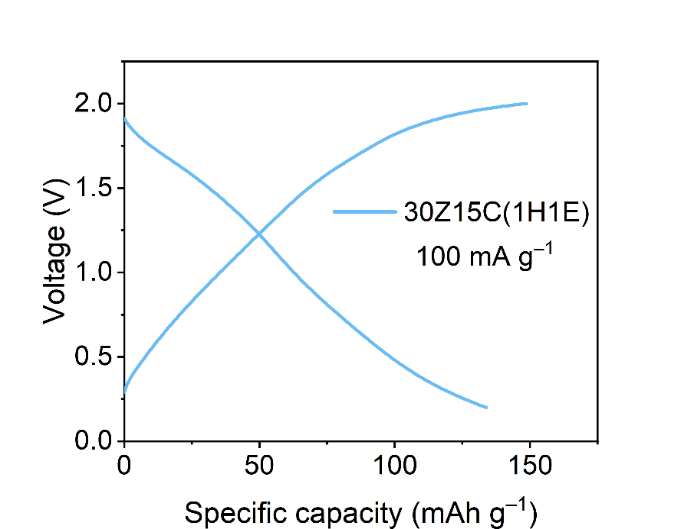


**Figure S3.** GCD profile of the Zn||AC battery in 30Z15C(1H1E) electrolyte at a current rate of 100 mA g^–1^.


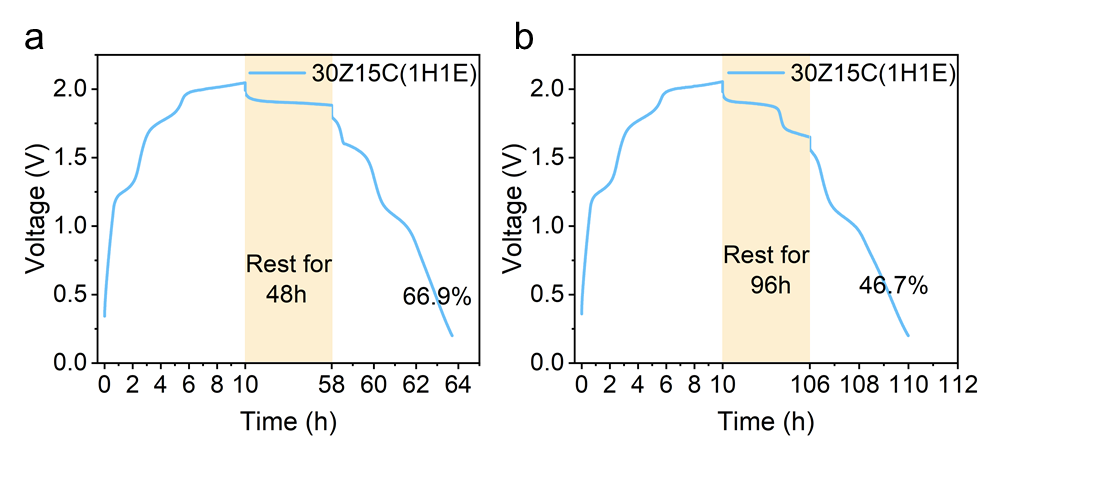


**Figure S4**. Voltage profiles of Zn||I_2_ batteries with (a) 48h rest and (b) 96h between charge and discharge showed 66.9% and 46.7% capacity retention for the 30Z15C(1H1E).


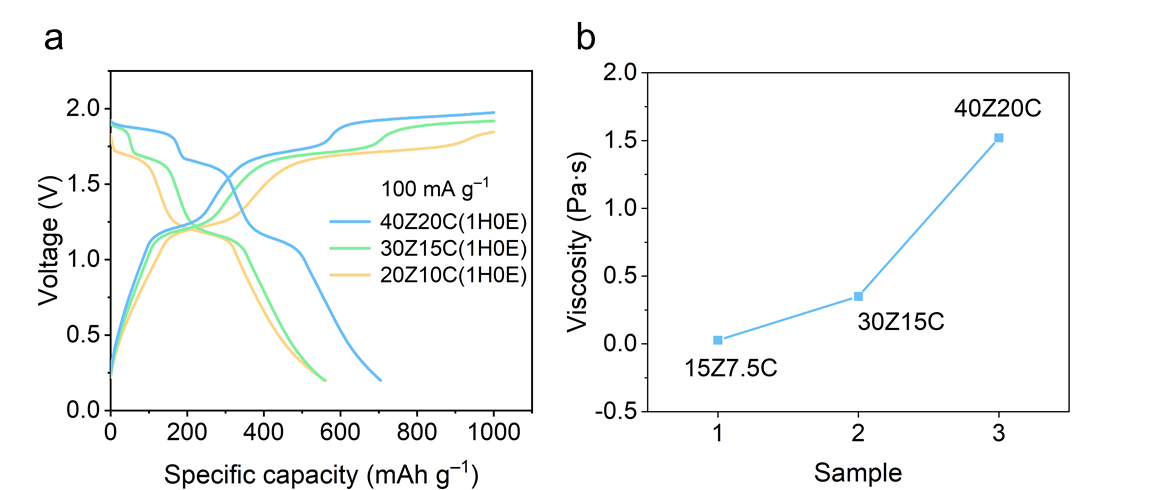


**Figure S5**. (a) GCD profiles of Zn||I_2_ batteries in 20Z10C(1H0E), 30Z15C(1H0E), and 40Z20C(1H0E) electrolytes at a current rate of 100 mA g^–1^. (b) Viscosity corresponds to the three electrolytes.


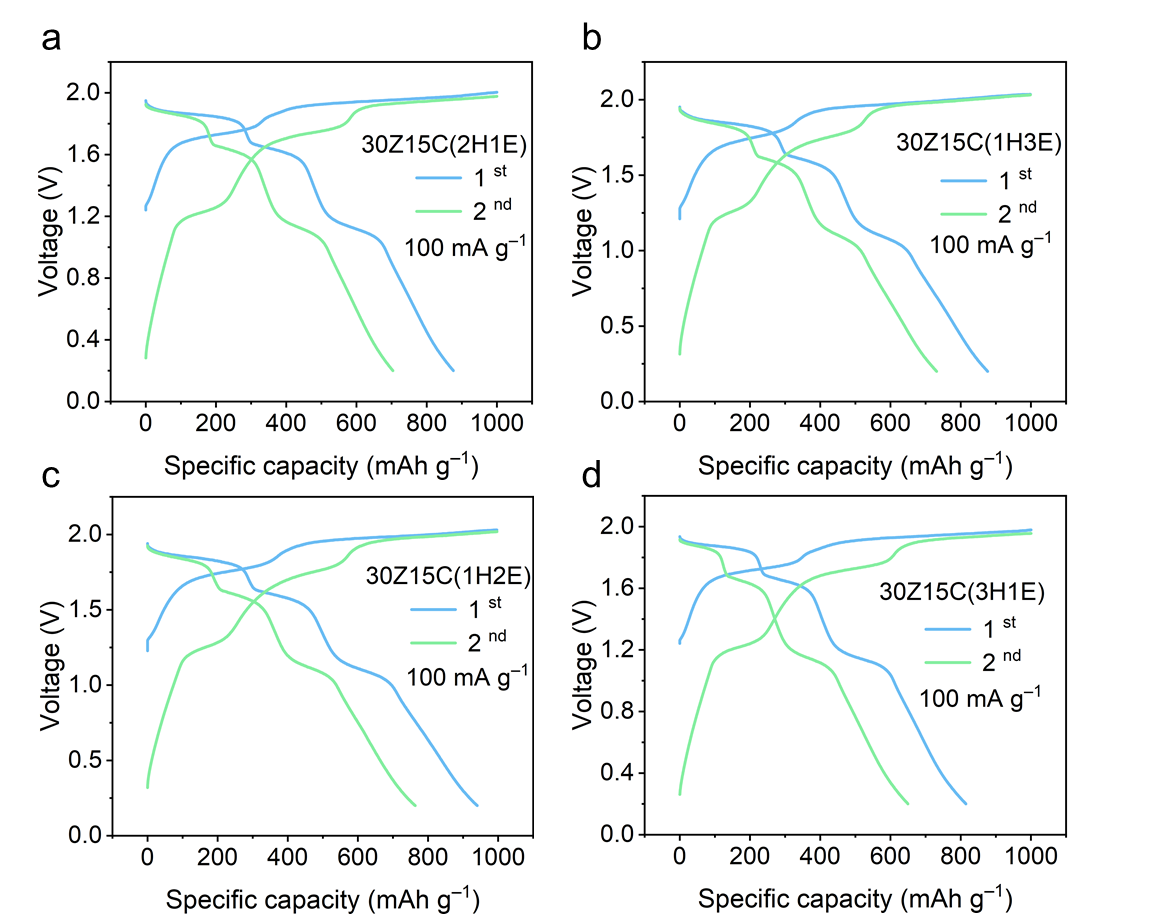


**Figure S6**. GCD profiles of Zn||I_2_ batteries in the (a) 30Z15C(3H1E), (b) 30Z15C(2H0E), (c) 30Z15C(1H2E), and (d) 30Z15C(1H3E) electrolytes, respectively.


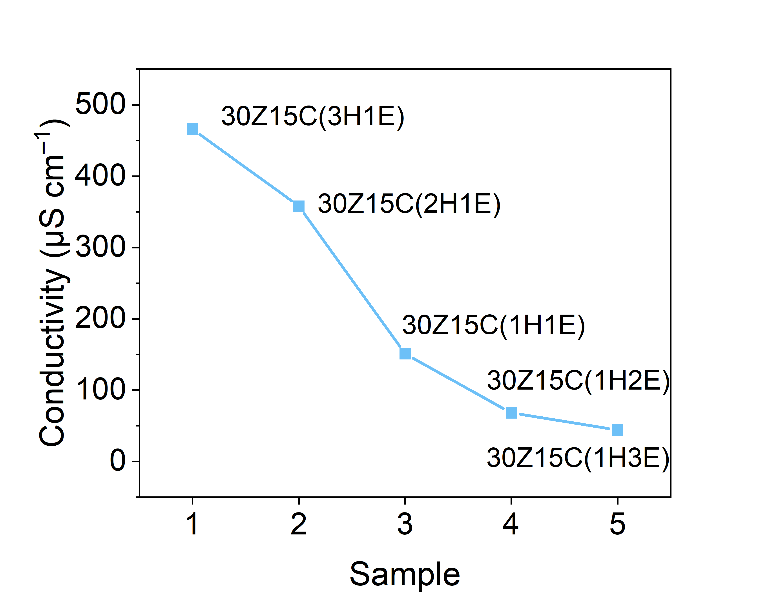


**Figure S7**. Conductivity of the 30Z15C(3H1E), 30Z15C(2H1E), 30Z15C(1H1E), 30Z15C(1H2E), and 30Z15C(1H3E) electrolytes.


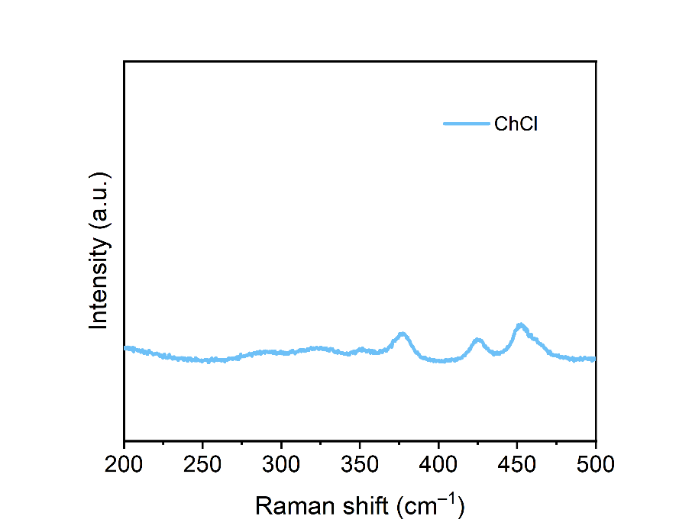


**Figure S8**. Raman spectrum of ChCl within 200–500 cm^–1^.


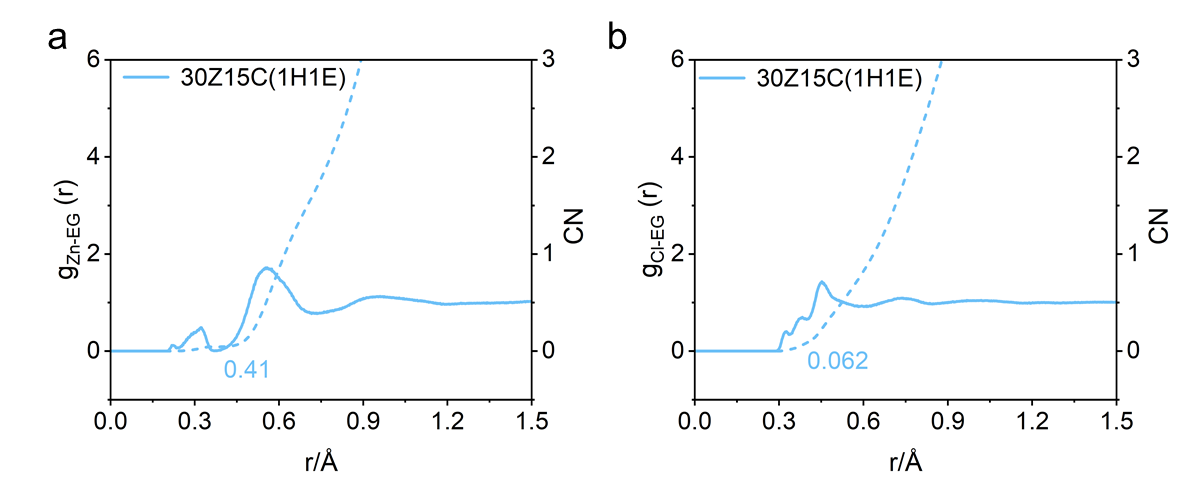


**Figure S9**. Radical distribution function (RDF, solid lines) and coordination numbers (CN, dotted lines) of (a) Zn-EG and (b) Cl-EG in the 30Z15C(1H1E).


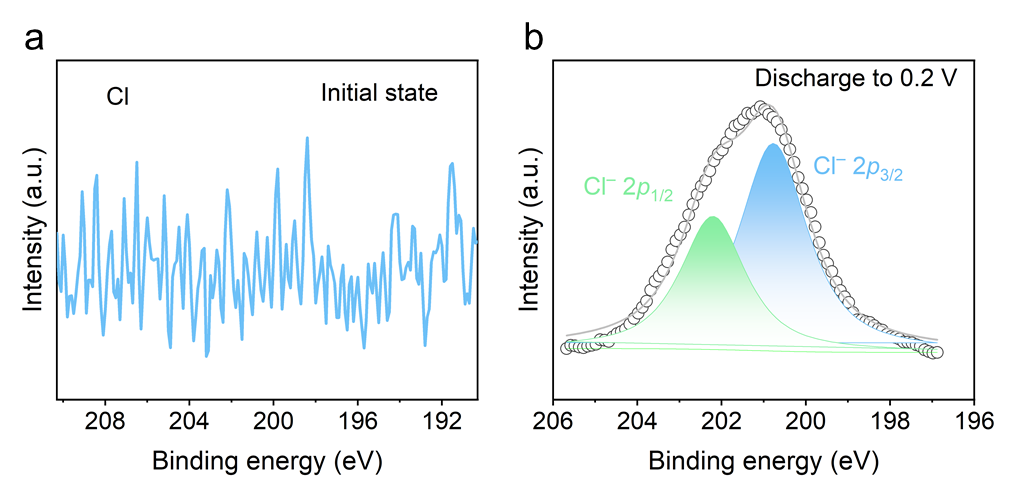


**Figure S10**. XPS spectra of Cl in SOCs of (a) initial state and (b) discharge to 0.2 V state for the I_2_/AC cathode.


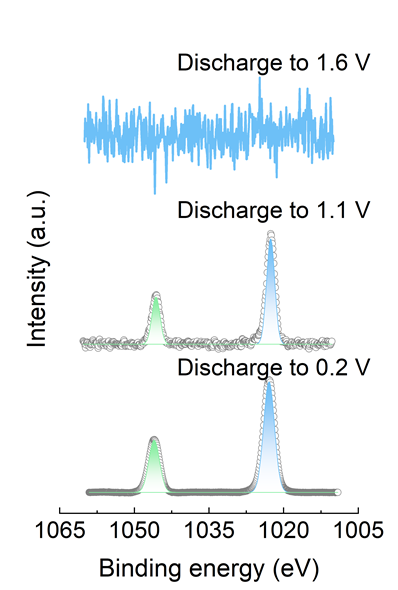


**Figure S11**. XPS spectra of Zn in I_2_/AC cathode at different SOCs.


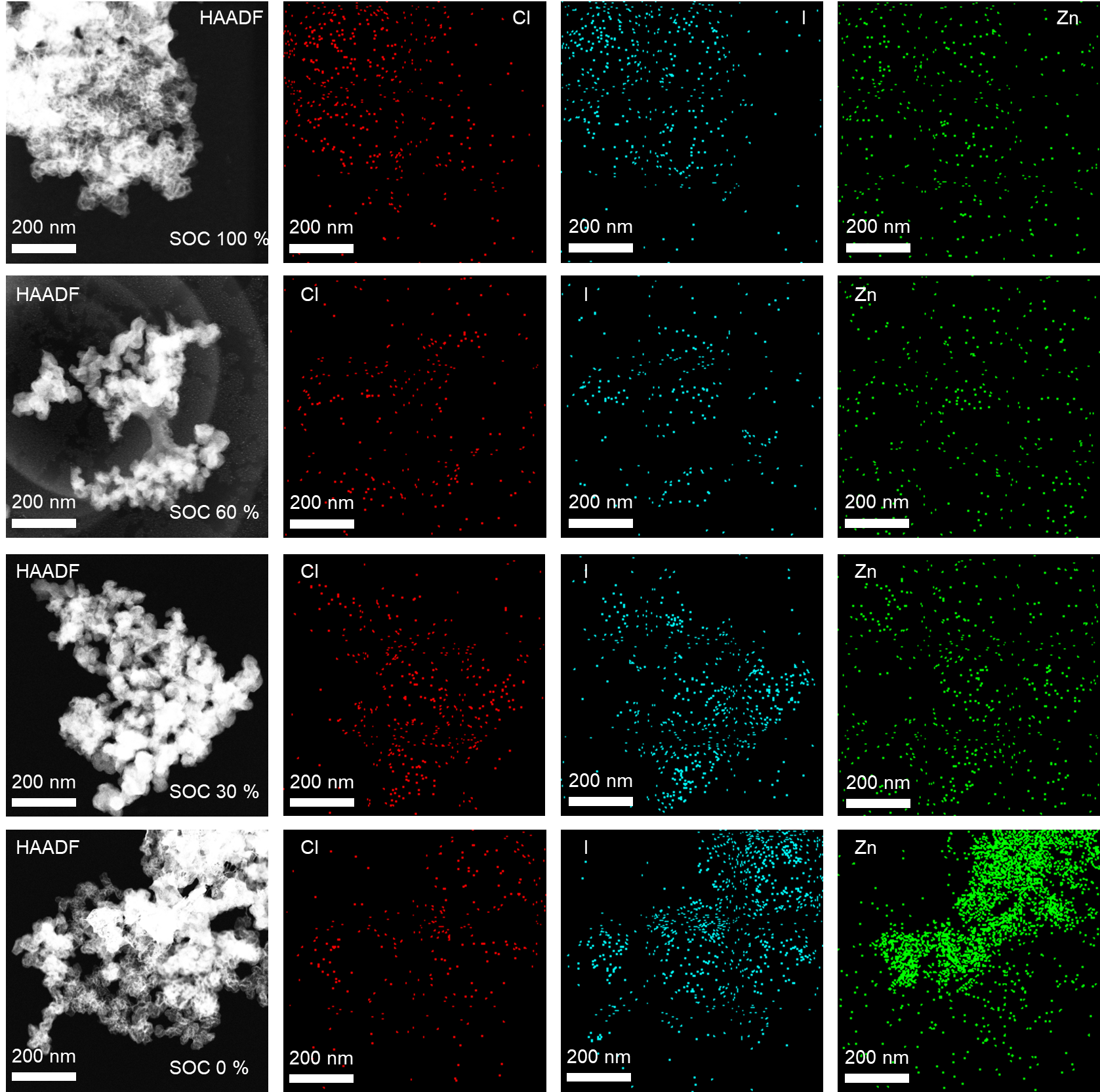


**Figure S12**. HAADF-STEM images of I_2_/AC cathode at different SOCs and the corresponding EDX elemental mappings of Cl, I, and Zn elements.


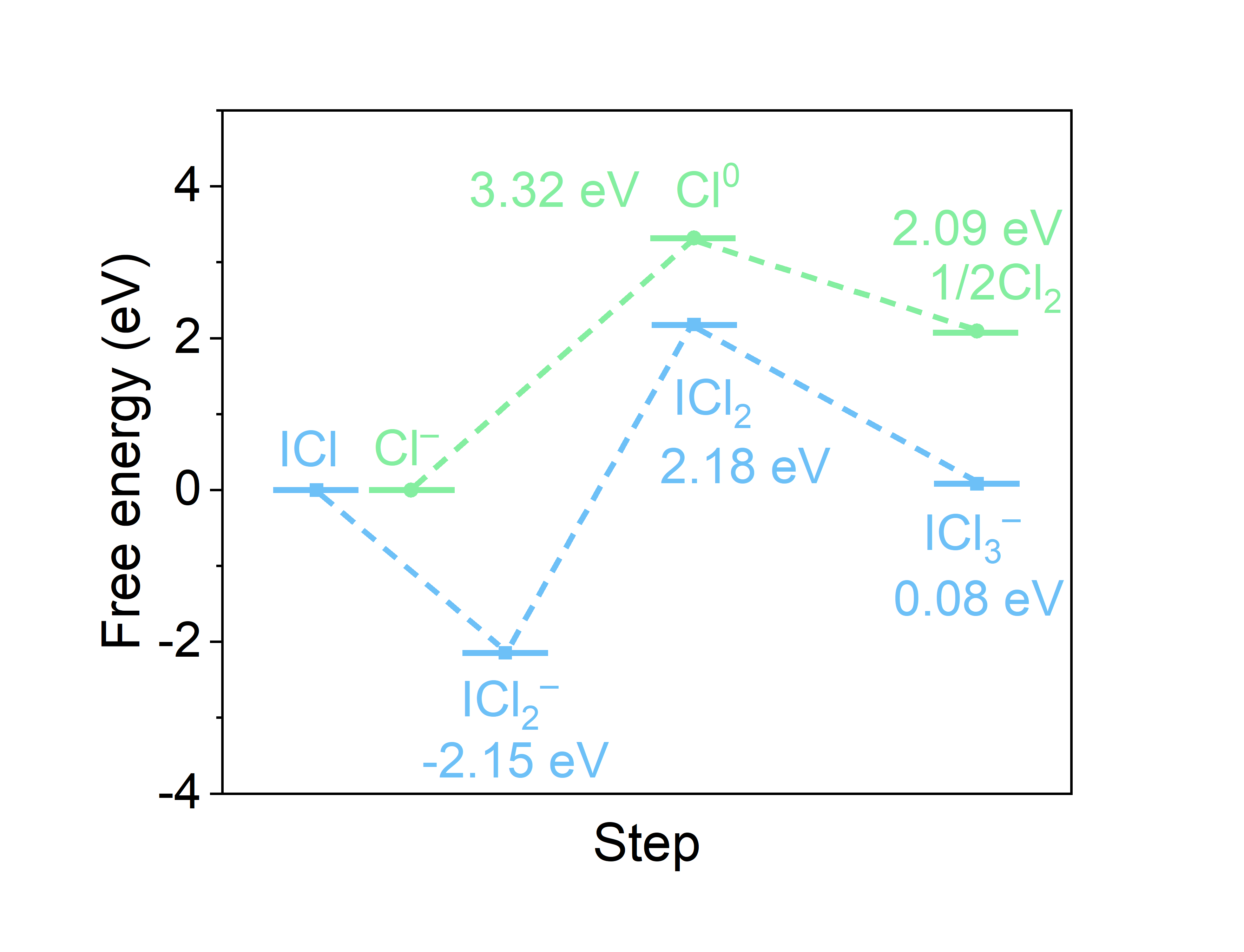


**Figure S13**. Energy profiles of ICl reaction pathways with different corresponding products.


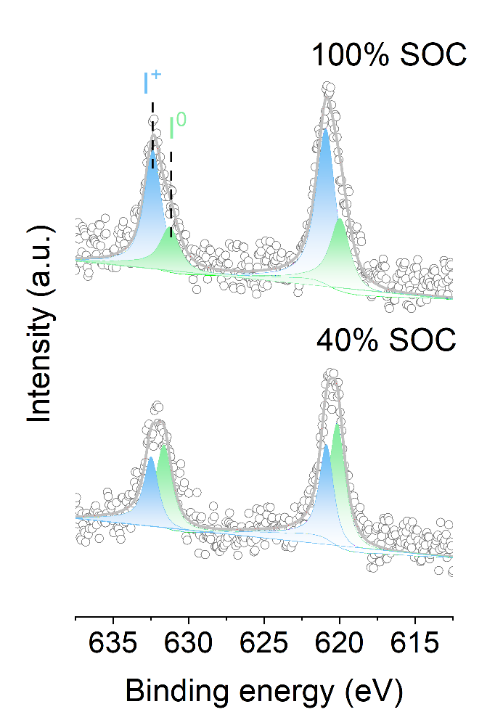


**Figure S14**. XPS spectra of I in the subsequent charged cycle.


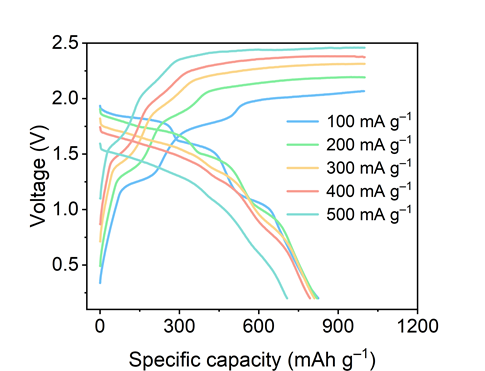


**Figure S15**. Rate performance of the Zn||I_2_ battery in the 30Z15C(1H1E) electrolyte.


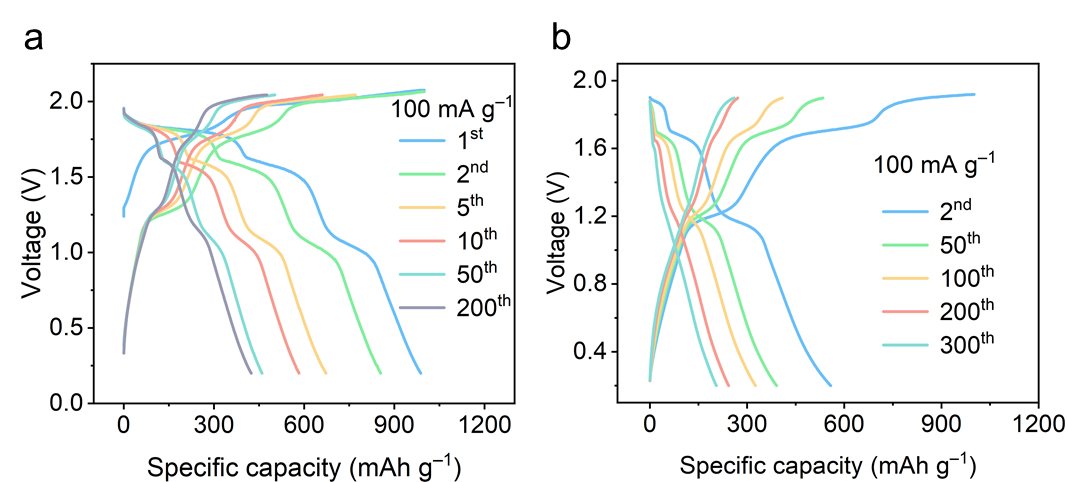


**Figure S16**. GCD profiles of Zn||I_2_ batteries in the (a) 30Z15C(1H1E) electrolyte and (b) 30Z15C(1H0E) electrolyte.


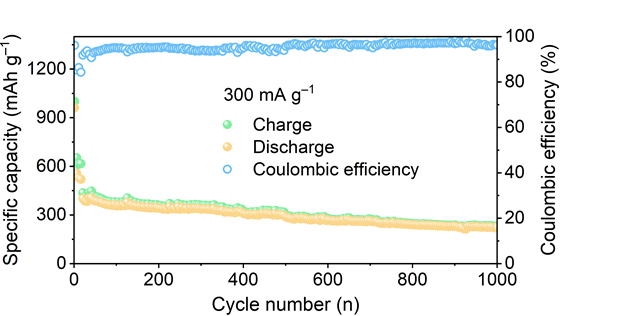


**Figure S17**. Long-term cycling at a current rate of 300 mA g^−1^ of the Zn||I_2_ battery in the 30Z15C(1H1E) electrolyte.

**Table S1.** The percentage of main ion species bonded to Zn^2+^ in the 30Z15C(1H1E) electrolyte according to MD simulation.

| Species | | | | Apparency | Content |
| --- | --- | --- | --- | --- | --- |
| Cl | ChCl | H_2_O | EG |  |  |
| 3 | 0 | 1 | 0 | 181 | 30.17% |
| 4 | 0 | 0 | 0 | 133 | 22.17% |
| 4 | 1 | 0 | 0 | 35 | 5.83% |
| 3 | 1 | 1 | 0 | 34 | 5.67% |
| 3 | 0 | 0 | 0 | 32 | 5.33% |
| 2 | 0 | 1 | 0 | 20 | 3.33% |
| 4 | 0 | 1 | 0 | 17 | 2.83% |
| 2 | 0 | 0 | 0 | 16 | 2.67% |
| 3 | 0 | 2 | 0 | 15 | 2.50% |
| 3 | 0 | 1 | 1 | 13 | 2.17% |
| 6 | 0 | 0 | 0 | 10 | 1.67% |
| 1 | 0 | 1 | 0 | 10 | 1.67% |
| 3 | 0 | 0 | 1 | 10 | 1.67% |
| 2 | 0 | 4 | 0 | 8 | 1.33% |
| 4 | 2 | 0 | 0 | 7 | 1.17% |
| 4 | 1 | 1 | 0 | 7 | 1.17% |
| 4 | 0 | 0 | 1 | 7 | 1.17% |
| 2 | 0 | 2 | 0 | 6 | 1.00% |
| 3 | 1 | 0 | 0 | 5 | 0.83% |
| 2 | 0 | 0 | 1 | 4 | 0.67% |
| 3 | 2 | 1 | 0 | 4 | 0.67% |
| 3 | 1 | 0 | 1 | 3 | 0.50% |
| 2 | 0 | 2 | 1 | 3 | 0.50% |

**Table S2**. Composition of the I and Cl in various valence states at different SOCs as determined by fitting XPS data.

| Species | Content in atomic percentage (%) | | | | |
| --- | --- | --- | --- | --- | --- |
|  | 100%SOC | 75%SOC | 50%SOC | 25%SOC | 0%SOC |
| I^+^ | 71.7 | 53.7 | 43.8 | / | / |
| I^0^ | 28.3 | 46.3 | 56.2 | 64.8 | 33.5 |
| I^–^ | / | / | / | 35.2 | 66.5 |
| Cl^0^ | 62.7 | 57.0 | / | / | / |
| Cl^–^ | 37.3 | 43.0 | 100 | 100 | 100 |

**Table S3**. The elemental analysis of the I_2_/AC electrode at different SOCs from STEM measurements.

| SOCs | Element | Mass% | Atom% | Chemical formula |
| --- | --- | --- | --- | --- |
| 100% | Cl | 0.56 | 0.19 | ICl_2.38_ |
|  | I | 0.85 | 0.08 |  |
| 60% | Cl | 0.11 | 0.04 | ICl_1.33_ |
|  | I | 0.33 | 0.03 |  |
| 30% | Cl | 0.27 | 0.09 | ICl_0.64_ |
|  | I | 1.40 | 0.14 |  |
| 0% | Cl | 0.11 | 0.08 | ICl_0.25_ |
|  | I | 1.62 | 0.32 |  |

**Table S4**. Internal resistance, charge transfer resistance, and fitted diffusion coefficients of Zn||I_2_ batteries at different SOCs.

| SOCs | Internal resistance  (R_Ω_, Ω) | Charge transfer resistance (R_ct_, Ω) | Diffusion coefficient (lgD, cm^–2^ s^–1^) |
| --- | --- | --- | --- |
| 100% | 140.0 | 120.9 | -13.8313 |
| 50% | 129.6 | 87.1 | -13.7112 |
| 25% | 116.7 | 75.9 | -13.4532 |
| 0% | 83.3 | 68.2 | -12.8495 |

**Table S5**. Representative electrochemical performance comparison of Zn||I_2_ batteries in different systems.

| Cathode | Electrolyte | Capacity (mAh g^–1^) | Energy  density  (Wh kg^–1^) | Cycle number | Reference |
| --- | --- | --- | --- | --- | --- |
| I_2_/Activated carbon | **30Z15C(1H1E)** | **987** | **1278** | **1000** | **This work** |
| Ti_3_C_2_I_2_ MXene | 2 m ZnCl_2_ + 1 m KCl | 207 | 280 | 2800 | [1] |
| Activated carbon cloth/iodine | 1 M ZnSO_4_ | 255 | 151 | 1500 | [2] |
| Ketjen black | Catholyte: 0.5 M ZnSO_4_ + 1 M LiI + 0.1 M I_2,_ Anolyte: 0.5 M ZnSO_4_ + 0.5 M Li_2_SO_4_ | 201 | 100 | 6000 | [3] |
| Host-nitrogen-doped porous carbon nanocages/I_2_ | 2 M ZnSO_4_ | 259 | 282 | 3500 | [4] |
| I_2_@N-doped hierarchical porous carbon | 1 M ZnSO_4_ | 219 | 72.6 | 10000 | [5] |
| I_2_@activated carbon | 2 M Zn(CF_3_SO_3_)_2_ | 210 | 237 | 10000 | [6] |
| BiI_3_@porous hard carbon | 2 M Zn(CF_3_SO_3_)_2_ | 182 | / | 20000 | [7] |
| PAC-I_2_ | 14.91 m ZnCl_2_ + 14.91 m LiCl + 6.28 m ACN | 594 | 750 | 6000 | [8] |
| I_2_@graphitic/porous carbon microtubes | 1 M ZnSO_4_ + 0.1 M ZnI_2_ | 285 | 182 | 1000 | [9] |
| I_2_@porous carbon | 2 m ZnSO_4_ in propylene glycol: water = 5: 1 | 210 | / | 2000 | [10] |
| Activated carbon@I_2_ | 2 M ZnSO_4_ | 205 | 188 | 1500 | [11] |
| Three-dimensional carbon@I_2_ | 4 M ZnSO_4_ + 0.4 M LiI | 0.6 mAh cm^–2^ | 211 | 5000 | [12] |
| I_2_@Activated carbon | 2.7 m ZnSO_4_ | 173 | / | 2500 | [13] |
| I_2_@C | niacinamide: dimethyl sulfone: Zn(ClO_4_)_2_·6H_2_O = 0.5: 3: 1 | 412 | 404 | 2000 | [14] |
| Cationic cellulose nanofiber/Activated carbon@I2 | 2 M ZnSO_4_ | 182 | 34 | 3000 | [15] |
| I_2_@Activated carbon | 2 M ZnSO_4_ | 175 | 210 | 600 | [16] |
| I_2_/porous Prussian blue@polypyrrole | 2 M ZnSO_4_ | 248 | 215 | 10000 | [17] |
| CMK-3@I_2_ | 2 M ZnSO_4_ | 116 | 150 | 39000 | [18] |
| I_2_@Activated carbon | 2 mol L^–1^ ZnSO_4_ + 500 mmoL L^–1^ trimethylamine hydrochloride | 450 | / | 5000 | [19] |
| Trimethylamine hydrochloride/I_2_ | 2 M ZnSO_4_ + 25 mM ZnI_2_ | 261 | / | 3500 | [20] |
| I_2_/active carbon fiber | 1 M Zn(ClO_4_)_2_-ACN | 200 | / | 7000 | [21] |
| Carbon nanotubes/ I_2_ | polyanionic hydrogel + polycationic hydrogel/ cellulose paper immersing in 2 M ZnSO_4_ | 220 | / | 18000 | [22] |
| Activated carbon@I_2_ | PEO/PVDF/CD-Si | 144 | / | 35000 | [23] |
| Activated carbon | ZnI_2_ + I_2_ + 3 M Zn(OTf)_2_ | 130 | / | 1000 | [24] |
| Super P | pyridine-2 M ZnSO_4_ | 180 | / | 25000 | [25] |
| Starch | Catholyte: 1 M LiI + 0.1 M I_2_  Anolyte: 0.5 M ZnSO_4_ + 0.5 M Li_2_SO_4_ | 182 | 80 | 50000 | [26] |
| In-MOF | 3 M Zn(CF_3_SO_3_)_2_ + 1 M 1-methyl-3-propylimidazolium iodine | 481 | 566 | 2500 | [27] |
| Carbon cloth | Catholyte: F77 block copolymer (35 wt%) + 0.1 m I_2_ + 1 m KI  Anolyte: F77 block copolymer (35 wt%) + 0.5 m ZnSO4 | 252 | / | 500 | [28] |
| I_2_@Activated carbon | 30 m ZnCl_2_ | 612 | 905 | 2000 | [29] |
| I_2_/HAC | 0.1 M H_2_SO_4_ + 0.1 M KBr | 1200 | 1357 | 150 | [30] |
| PAC/I_2_ | 3 M ZnSO_4_ + 1 M ZnCl_2_ | 445 | / | 2000 | [31] |

M represents the volume molar concentration with units of mol L^–1^, and m represents the mass molar concentration with units of mol kg^–1^.

**References**

[1] X. Li, M. Li, Z. Huang, G. Liang, Z. Chen, Q. Yang, Q. Huang, C. Zhi, *Energy Environ. Sci.* **2021**, *14*, 407.

[2] C. Bai, F. Cai, L. Wang, S. Guo, X. Liu, Z. Yuan, *Nano Res.* **2018**, *11*, 3548.

[3] H. J. Yang, Y. Qiao, Z. Chang, H. Deng, P. He, H. S. Zhou, *Adv. Mater.* **2020**, *32*, e2004240.

[4] W. Liu, P. Liu, Y. Lyu, J. Wen, R. Hao, J. Zheng, K. Liu, Y.-J. Li, S. Wang, *ACS Appl. Mater. Interfaces* **2022**, *14*, 8955.

[5] Z. Gong, C. Song, C. Bai, X. Zhao, Z. Luo, G. Qi, X. Liu, C. Wang, Y. Duan, Z. Yuan, *Sci. China Mater.* **2022**, *66*, 556.

[6] W. Li, K. Wang, K. Jiang, *J. Mater. Chem. A* **2020**, *8*, 3785.

[7] Q. Deng, F. Liu, X. Wu, C. Li, W. Zhou, B. Long, *J. Energy Chem.* **2024**, *89*, 670.

[8] Y. P. Zou, T. T. Liu, Q. J. Du, Y. Y. Li, H. B. Yi, X. Zhou, Z. X. Li, L. J. Gao, L. Zhang, X. Liang, *Nat. Commun.* **2021**, *12*, 170.

[9] S. Chai, J. Yao, Y. Wang, J. Zhu, J. Jiang, *Chem. Eng. J.* **2022**, *439*, 135676.

[10] J. Hao, L. Yuan, Y. Zhu, X. Bai, C. Ye, Y. Jiao, S. Z. Qiao, *Angew. Chem. Int. Ed.* **2023**, *62*, e202310284.

[11] J.-L. Yang, H.-H. Liu, X.-X. Zhao, X.-Y. Zhang, K.-Y. Zhang, M.-Y. Ma, Z.-Y. Gu, J.-M. Cao, X.-L. Wu, *J. Am. Chem. Soc.* **2024**, *146*, 6628.

[12] Q. Chen, S. Chen, J. Zhang, *J. Power Sources* **2023**, *556*, 232529.

[13] H. Wang, X. Liu, J. Zhong, L. Du, S. Yun, X. Zhang, Y. Gao, L. Kang, *Small* **2024**, *20*, 2306947.

[14] W. Li, H. Xu, H. Zhang, F. Wei, T. Zhang, Y. Wu, L. Huang, J. Fu, C. Jing, J. Cheng, S. Liu, *Energy Environ. Sci.* **2023**, *16*, 4502.

[15] Z. Li, W. Cao, T. Hu, Y. Hu, R. Zhang, H. Cui, F. Mo, C. Liu, C. Zhi, G. Liang, *Angew. Chem. Int. Ed.* **2024**, *63*, e202317652.

[16] K. K. Sonigara, J. V. Vaghasiya, M. Pumera, *Adv. Energy Mater.* **2024**, 2401321.

[17] M. Wang, J. Ma, H. Zhang, L. Fu, X. Li, K. Lu, *Small* **2024**, *20*, 2307021.

[18] Q. Guo, H. Wang, X. Sun, Y. n. Yang, N. Chen, L. Qu, *ACS Mater. Lett.* **2022**, *4*, 1872.

[19] M. Wang, Y. Meng, M. Sajid, Z. Xie, P. Tong, Z. Ma, K. Zhang, D. Shen, R. Luo, L. Song, L. Wu, X. Zheng, X. Li, W. Chen, *Angew. Chem. Int. Ed.* **2024**, *63*, e202404784.

[20] S. Niu, B. Zhao, D. Liu, *ACS Appl. Mater. Interfaces* **2023**, *15*, 25558.

[21] C. Song, Z. Gong, C. Bai, F. Cai, Z. Yuan, X. Liu, *Nano Res.* **2021**, *15*, 3170.

[22] J. L. Yang, Z. Yu, J. Wu, J. Li, L. Chen, T. Xiao, T. Xiao, D. Q. Cai, K. Liu, P. Yang, H. J. Fan, *Adv. Mater.* **2023**, *35*, 2306531.

[23] Y. Su, X. Wang, M. Zhang, H. Guo, H. Sun, G. Huang, D. Liu, G. Zhu, *Angew. Chem. Int. Ed.* **2023**, *62*, e202308182.

[24] K. Zhang, Q. Yu, J. Sun, Z. Tie, Z. Jin, *Adv. Mater.* **2024**, *36*, 2309838.

[25] Y. Lyu, J. A. Yuwono, P. Wang, Y. Wang, F. Yang, S. Liu, S. Zhang, B. Wang, K. Davey, J. Mao, Z. Guo, *Angew. Chem. Int. Ed.* **2023**, *135*, e202303011.

[26] S. J. Zhang, J. Hao, H. Li, P. F. Zhang, Z. W. Yin, Y. Y. Li, B. Zhang, Z. Lin, S. Z. Qiao, *Adv. Mater.* **2022**, *34*, 2201716.

[27] W. Du, L. Miao, Z. Song, X. Zheng, C. Hu, Y. Lv, L. Gan, M. Liu, *Chem. Eng. J.* **2024**, *484*, 149535.

[28] K. K. Sonigara, J. Zhao, H. K. Machhi, G. Cui, S. S. Soni, *Adv. Energy Mater.* **2020**, *10*, 2001997.

[29] G. Liang, B. Liang, A. Chen, J. Zhu, Q. Li, Z. Huang, X. Li, Y. Wang, X. Wang, B. Xiong, X. Jin, S. Bai, J. Fan, C. Zhi, *Nat. Commun.* **2023**, *14*, 1856.

[30] W. Ma, T. Liu, C. Xu, C. Lei, P. Jiang, X. He, X. Liang, *Nat. Commun.* **2023**, *14*, 5508.

[31] P. Jiang, Q. Du, C. Lei, C. Xu, T. Liu, X. He, X. Liang, *Chem. Sci.* **2024**, *15*, 3357.
